# A Subset of Colon Cancer Cell Lines Displays a Cytokine Profile Linked to Angiogenesis, EMT and Invasion Which Is Modulated by the Culture Conditions In Vitro

**DOI:** 10.3390/cells12212539

**Published:** 2023-10-29

**Authors:** Jacqueline Bersano, Kanstantsin Lashuk, Anna Edinger, Julia Schueler

**Affiliations:** Charles River Discovery Research Services Germany GmbH, Am Flughafen 12–14, 79108 Freiburg, Germany; jacqueline.bersano@crl.com (J.B.); kanstantsin.lashuk@crl.com (K.L.); anna.edinger@crl.com (A.E.)

**Keywords:** colorectal cancer, cytokine profile, tumor microenvironment

## Abstract

Colorectal cancer (CRC) is one of the deadliest cancers worldwide. The dysregulation of secretory pathways is a crucial driver of CRC progression, since it modulates cell proliferation, angiogenesis and survival. This study explores the changes in the CRC cytokine profile depending on the culture conditions and the presence of fibroblasts and macrophages as cellular components of the tumor microenvironment in 2D and in 3D formed spheroids. Upon analysis of 45 different cytokines, chemokines and growth factors, 20 CRC cell lines were categorized into high and low secretors. In the high secretor group cytokines related to angiogenesis, EMT and invasion were significantly upregulated. LIF and HFG were identified as the best discriminator between both groups. Independent of this grouping, the addition of normal as well as cancer-associated fibroblasts had a similar impact on the cytokine profile by increasing the total amount of secreted cytokines in most of the investigated cell lines. In contrast, the differentiation and polarization of macrophages was modulated differently by normal vs. cancer-associated fibroblasts. In summary, we identified two groups of CRC cell lines that differ in their cytokine profile. The dependance of this profile was analyzed in detail—not only from the tumor cell line but as well from the culture condition in vitro. Key cytokines that discriminate the two groups were identified and their importance as promising biomarker candidates for CRC discussed.

## 1. Introduction

Colorectal cancer (CRC) represents the second leading cause of cancer death globally due to the high occurrence of metastasis in patients [1]. The dysregulation of the secretome is a major driver of the metastatic cascade in CRC due to its implication in epithelial-to-mesenchymal transition (EMT) induction, angiogenesis, migration and cell survival [2,3]. The secretome reflects the functionality of the cells since it comprises all the proteins released by a cell, organism or tissue, such as cytokines, growth factors, receptors and proteases and includes also the extracellular matrix components. Therefore, the cancer secretome constitutes a valuable source of prognostic and predictive biomarkers [4].

Besides identifying individual signaling cascades, an in-depth investigation of the main regulators of the secretome expression and their interaction becomes essential. Within the secretome, cytokines play an important role, as they also interact with non-cancerous cells and as a link between different organ systems [5]. Cancer cells do not induce dysregulated cytokine secretion alone, but rather create a bi-directional tumor–stroma signaling in the tumor microenvironment (TME) [6,7].

Stromal fibroblasts and macrophages represent the key components of the TME. Fibroblasts are the most abundant cells in the TME, they participate in the production of collagens and fibronectin, considered essential to cancer cell proliferation, migration and invasion [8]. Furthermore, fibroblasts secrete growth factors and cytokines that strongly influence the recruitment of immune cells and EMT processes, by promoting angiogenesis, tumor growth and a tumor-promoting, but immune-suppressive, microenvironment [9,10].

Likewise, the differentiation and polarization of monocytes into tumor-associated macrophages (TAMs) promote tumorigenesis and facilitate tumor growth and metastasis [11]. The phenotype of macrophages in normal tissues has been recognized as significantly different than in CRC at different stages. Several studies have demonstrated the polarization of macrophages in the later stages of the disease from a pro-inflammatory “M1” toward an anti-inflammatory phenotype, commonly called “M2-like” TAMs, which can drive tumorigenesis by secreting pro-metastatic cytokines and release metalloproteases [12,13]. Furthermore, recent findings have reported the ability of macrophages to differentiate into intermediates between the two states, highlighting the necessity of accurately examining the macrophage phenotype in CRC [14].

In the current study we aim to understand the bi-directional cross-talk of the different cellular components in CRC tumor tissue by focusing on the cytokine profile as important part of the secretome. By deconvoluting the different parameters the suitability of cytokine profiles as well as individual cytokines as possible biomarkers will be discussed.

## 2. Materials and Methods

### 2.1. Cell Culture

Human colorectal cancer (CRC) cell lines LS174T, RKO, COLO 205, Caco-2, HCT-15, DiFi, Lovo were obtained from the American Type Culture Collection (ATCC) (Rockville, MD, USA); HCT-116, HT-29, DLD-1, HCC-2998, KM12, KM20L2, SW-620, SW-480 from the National Cancer Institute (NCI); CRC cell lines 269, 94, 1103, 243, 280 (PDX derived cell lines) were established at Charles River from colon carcinoma patients at the University Hospital/University of Freiburg [15]. The identity of all cell lines was confirmed by STR analysis. Cells were cultured in Roswell Park Memorial Institute 1640 Medium (RPMI) (Anprotec, Bruckberg, Germany) containing 4 mM L-glutamine supplemented with 5% (*v*/*v*) fetal bovine serum (FBS) (Sigma Aldrich, Darmstadt, Germany) and 0.1% (*v*/*v*) gentamicin (GIBCO-BRL, Grand Island, NY, USA).

Human dermal fibroblasts (HDFs) were provided by ScienCell Research Laboratories (Carlsbad, CA, USA) and cultured in Fibroblast Growth Medium 3 (Promocell, Heidelberg, Germany) supplemented with 1% (*v*/*v*) of amphotericin B (Sigma-Aldrich, Darmstadt, Germany). Primary cancer-associated fibroblasts (CAFs) were previously isolated from colon carcinomas of patients (gift of Dolznig lab, Medical University of Vienna, Vienna, Austria). In this study CAFs 6388/19 were used. CAFs were cultured in endothelial growth medium (EGM-2MV, PromoCell, Germany). Peripheral blood mononuclear cells (PBMCs) were isolated from human peripheral blood of healthy donors. Cells were maintained at 37 °C and 5% CO_2_.

### 2.2. Isolation and Differentiation of Human Monocytes

Leukoreduction system (LRS) chambers were provided by the local blood bank and used as source of viable human peripheral blood mononuclear cells (PBMCs).

PBMCs isolation was executed via an adapted protocol of Néron et al. [16]. Three washing steps followed by a centrifugation cycle at 300× *g* for 5 min at room temperature (RT) were carried out. PBMC buffer (PBS containing 2 mM EDTA) was used in the first and last step, ammonium–chloride–potassium (ACK) lysing buffer in the second cycle. ACK lysing buffer was prepared as described by Brown et al. [17].

Human monocytes were isolated from PBMCs using the Monocyte Isolation Kit II (Miltenyi Biotec, Bergisch Gladbach, Germany) and frozen in Bambanker (Nippon Genetics, Dueren, Germany) at −80 °C. Monocytes were cultured in RPMI containing 4 mM L-glutamine, supplemented with 10% (*v*/*v*) FBS and 0.1% (*v*/*v*) gentamycin and differentiated into macrophages for 4 days with 20 ng/mL Granulocyte–macrophage colony-stimulating factor (GM-CSF) (Miltenyi Biotec, Germany).

### 2.3. 2D and 3D Spheroids Cultures

We cultured 1 × 10⁴ tumor cells in flat-bottom 96-well plates for the 2D setting, or ultra-low adhesion (ULA) round-bottom 96-well plates (Corning, Glendale, AZ, USA) to induce spheroid formation for 2 days in RPMI. Tumor cells were seeded in 200 µL of RPMI in mono-culture and 100 µL when in coculture with fibroblasts. To generate 3D coculture conditions 6 × 10^3^ HDFs or CAFs were seeded in 100 µL of RPMI together with the tumor cells on day 1 and incubated at 37 °C and 5% CO_2_. On day 3, 150 µL of cell culture medium were replaced with fresh medium. The above-mentioned cell numbers and cell culture medium volumes were used for the tested conditions (mono and coculture) in a consistent way across the different experiments. On day 6, 70 µL of cell supernatants were harvested, frozen at −20 °C and subsequently used for human specific magnetic bead-based assays or flow cytometry (FC) analysis. The culture duration was the same for all experiments and cell lines.

### 2.4. 3D Matrigel-Collagen Matrix

The modulation of cytokine secretion and macrophage differentiation was evaluated in a 3D spheroid culture setting with a matrigel–collagen matrix. A mixture of 4 mg/mL matrigel (Corning, NY, USA) and 3.39 mg/mL collagen (Collagen I, Rat Tail, Corning, NY, USA) matrix were prepared as follows: Matrigel was thawed overnight at 4 °C and then 10 mL were mixed with 121.2 µL 45% glucose (Merck-Millipore, Darmstadt, Germany), 252.5 µL Glutamax (GIBCO-BRL, Grand Island, NY, USA), 2.5 mL FBS and 1.23 mL RPMI in order to have a 4 mg/mL matrigel solution. 1.67 mL collagen I was mixed with 145 µL 10X RPMI, 59 µL 1M HEPES (GIBCO-BRL, Grand Island, NY, USA), 59 µL 7.5% Sodium Bicarbonate (Sigma-Aldrich, Darmstadt, Germany), 31.3 µL 1N NaOH (Fluka, Loughborough, UK) and 39 µL FBS. The final concentration of collagen was 3.39 mg/mL. Afterwards the matrigel matrix was mixed with a collagen matrix in a 2:7 collagen/5:7 matrigel ratio. Every step was performed on ice to avoid polymerization.

In this setting, at day 3 after spheroid formation in mono- or coculture with fibroblasts, 170 µL of the medium were replaced by 70 µL of a matrigel–collagen mixture. 5 × 10⁴ macrophages were resuspended in the matrigel–collagen mixture and added to the wells on day 3. The plate was incubated for 1 h at 37 °C to induce polymerization. 100 µL of cell culture medium were added on top of the polymerized mixture.

### 2.5. Flow Cytometry

Flow cytometry (FC) was used to analyze the polarization into M1-like and M2-like macrophages in mono-, co- and triple cultures with tumor cells and fibroblasts in a 3D matrigel–collagen matrix setting. Matrigel–collagen mix was digested as follows: 100 µL of cell culture medium were removed from the plate and 150 µL of Accutase (Sigma-Aldrich, Darmstadt, Germany) were added. The gel was mechanically disrupted after three cycles of mixing and incubation for 10 min at 4 °C. The replicates were then pooled together and centrifugated at 400× *g* for 5 min at 4 °C.

After digestion of the matrigel–collagen matrix, the supernatant was discarded, the cells washed twice with PBS and resuspended in 100 µL of live-dead staining solution (PBS supplemented with Zombie Aqua™ dye (Biolegend, San Diego, CA, USA), 1:100 dilution). After 30 min of incubation at 4 °C in the dark, cells were washed with PBS and stained for 30 min at 4 °C in 100 µL FACS buffer (PBS with 2% FCS) with the appropriate dilutions of antibodies for cell surface markers: CD163- PerCp/Cy5.5 (1:50), CD206-APC (1:50), CD45-BV421 (1:50), CD86-PE (1:20), CD11b-PE/Dazzle 594 (1:50), CD209- PE-Cy7 (1:50). After the incubation the cells were again washed in PBS and resuspended in 200 µL FACS buffer for FC analysis. Data were obtained with Attune NxT Flow Cytometer (Thermo Fisher Scientific, Waltham, MA, USA) and analyzed with FlowJo_V10. CD163, CD206, CD45, CD11b, CD209 antibodies were purchased from Biolegend (San Diego, CA, USA), CD86 from BD Biosciences (Franklin Lakes, NJ, USA).

### 2.6. Multiplex Bead Assay Analysis

The supernatant was collected and snap frozen. Cytokine analysis was performed on the thawed supernatant with “Cytokine/Chemokine/Growth Factor 45-Plex Human ProcartaPlex™ Panel 1” kit (Thermo Fisher Scientific, Waltham, MA, USA) used according to the protocol. Data were obtained with a Bio-Plex 200 Systems (BioRad, Hercules, CA, USA).

### 2.7. Incucyte Software Analysis

Incucyte^®^ S3 Live-Cell Analysis System was used to monitor spheroid formation. Images were taken every 6 h. The 2D monolayer assay doubling times (h) shown in Table S1 were used to calculate proliferation rates in 2D. To assess spheroid growth, the area under the curve (AUC) was calculated from the largest brightfield object areas (µm^2^) exported for each time point. Largest brightfield object areas (µm^2^) were measured with the use of the Incucyte^®^ S3 software version 2022C.

## 3. Results

### 3.1. Culturing Colorectal Cancer Cell Lines in 3D Spheroid Cultures and in Coculture with Fibroblasts Enhances the Secretion of Cytokines

**Figure 1:**
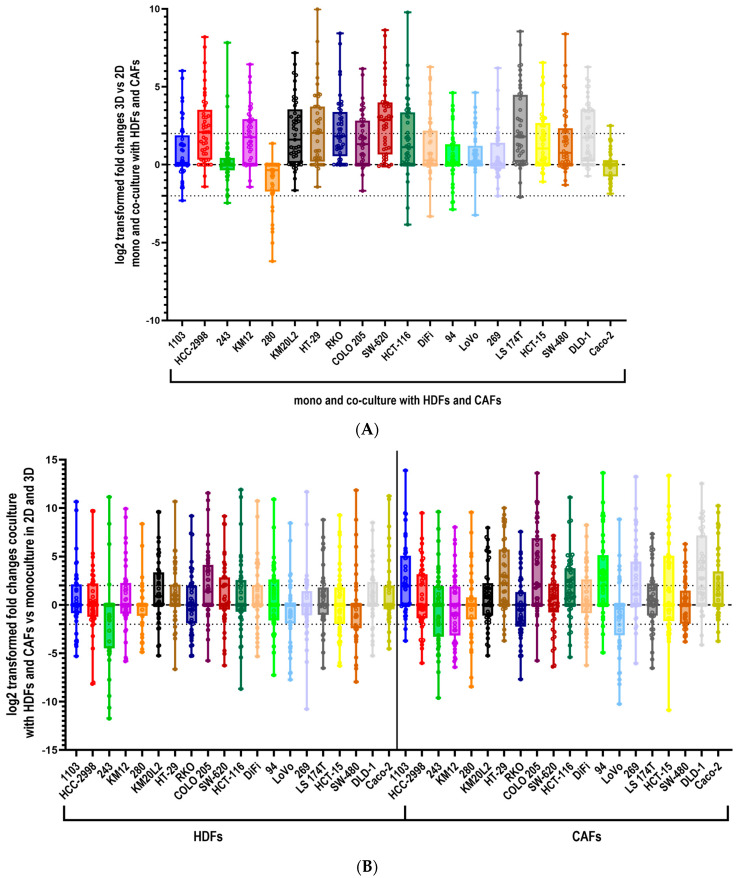
Cytokines secretion is positively regulated in 3D spheroid cultures and coculture with fibroblasts compared to 2D culture and monoculture. (**A**) Log2 transformed fold changes 3D vs. 2D monoculture and coculture with HDFs and CAFs. The graph shows pooled data from mono- and coculture with HDFs and CAFs to highlight the different cytokine expression between the 3D and 2D settings. (**B**) Log2 transformed fold changes coculture with HDFs and CAFs vs. monoculture in 2D and 3D. 1 × 10⁴ colon cancer cell lines (15 commercial lines and five PDX-derived) were cultured in 2D culture and 3D spheroids culture in monoculture and coculture with 6 × 10^3^ human dermal fibroblasts (HDF) and cancer-associated fibroblasts (CAFs). Supernatants were harvested on day 6 for the 3D culture and day 3 for the 2D culture setting. The expression of 45 cytokines was determined with a human-specific magnetic bead-based assay. The fold changes of the cytokine expression were calculated between 2D and 3D setting in mono- and coculture with HDFs and CAFs and between monoculture and coculture across 2D and 3D with both types of fibroblasts.

In a first attempt to explore the role of secreted factors in colon cancer, we analyzed the expression of 45 cytokines, chemokines and growth factors in the supernatant of 20 CRC cell lines cultivated in 2D, 3D spheroid monoculture and in coculture with HDFs and CAFs, respectively. In general, an upregulation of the cytokine secretion in 3D vs. 2D and in coculture vs. monoculture was observed (Figure 1A,B). We observed that 15 out of 20 cell lines displayed an upregulated cytokine secretion in 3D vs. 2D monoculture, 18 and 17 tumor cell lines secreted higher levels of cytokines in 3D compared to 2D when in coculture with HDFs and CAFs, respectively (Supplementary Figure S1A–C). Inversely to this observation, the PDX-derived cell lines (280, 269, 94 and 1103) and the publicly available line Caco-2 secreted more cytokines in 2D compared to 3D in monoculture (Figure S1A). CRC cell line 280 exhibited an upregulation of cytokine levels in 2D with both types of fibroblasts, while for Caco-2 and PDX-derived cell line 243 this was only the case in the coculture setting with CAFs (Figure S1B,C). When calculating the fold-changes between mono- and coculture in 2D and 3D the above-described results were confirmed (Figure S1D,E).

A detailed analysis of individual cytokine levels revealed that specifically cytokines with pro-inflammatory, pro-angiogenic and tumorigenic capabilities were elevated in 3D and coculture. Among them, EGF, HGF, PIGF-1, GM-CSF, IL-5, IL-9 and IL-6 were exclusively secreted in 3D and in coculture with HDFs and CAFs (Figure S2A).

### 3.2. Two Distinct Subgroups Were Identified Based on the Cytokine Profile In Vitro Displaying Differences in the Secretion of Cytokines Related to Angiogenesis, EMT and Invasion

**Figure 2:**
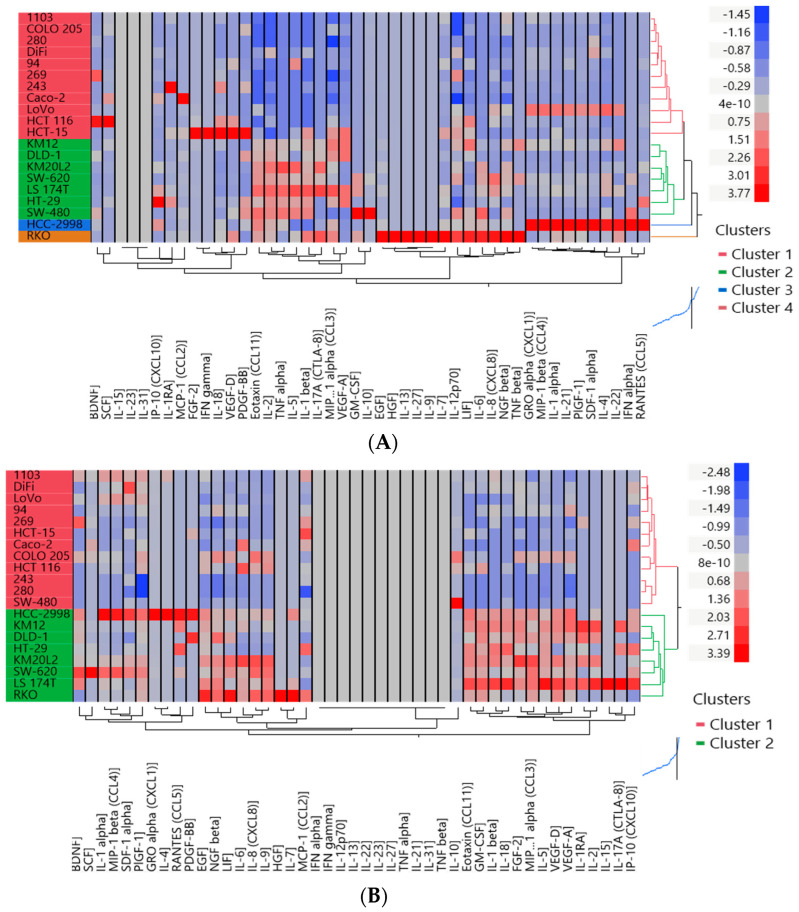

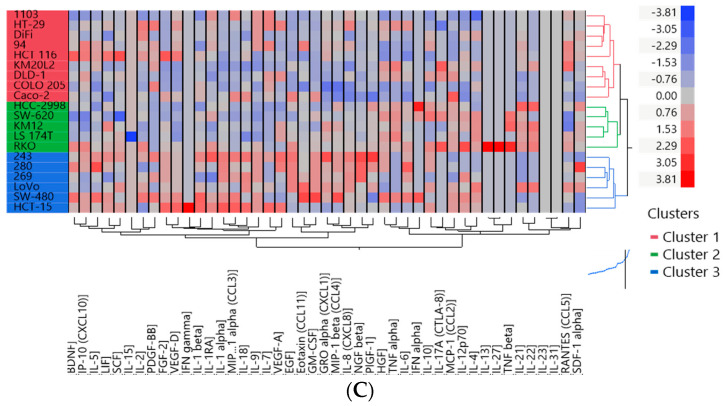
Cytokine profiles of 20 CRC cell lines in 3D spheroid cultures in monoculture and in coculture with HDFs. (**A**) Heat map of the cytokine levels (pg/mL) in 3D spheroids monoculture. (**B**) Heat map of the cytokine levels data (pg/mL) in 3D spheroids coculture with HDF. (**C**) Heat map of the fold changes 3D coculture with HDFs vs. 3D monoculture. Before clustering, (**A**,**B**) heatmap values were standardized together, (**C**) heatmap values were standardized separately. Hierarchical clustering (Method = Ward) is shown. n = 2.

A cluster analysis based on the monoculture 3D data displayed four clusters with two of them represented by one line each (HC-2998 and RKO, Figure 2A). The same analysis based on 3D coculture data (HDFs) exhibits two clusters with RKO and HCC-2988 in cluster 2 (Figure 2B). When analyzing the fold changes between those two settings a third cluster was identified including the lines secreting higher levels of cytokines in the monoculture setting (Figure 2C). A cluster analysis based on the results of the CAF- coculture in 3D confirmed the clustering into two and three clusters, respectively (Figure S2B,C).

To better understand the biological implications of those cluster analyses we allocated cell lines which were in at least two cluster analyses in cluster 1 and cluster 3 into group 1; and the cell lines which were in at least one part of cluster 2 into group 2 (Table 1, Figure 2A–C). The two groups differ significantly in the secretion of factors that are involved in angiogenesis (HGF, VEGF-A, VEGF-D), EMT-processes (IL-2, IL-17A, NGF-ß, IL-10, IL-8), cell proliferation and invasion (EGF, Eotaxin, GM-CSF, LIF) (Figure S2D). A partition plIt identified LIF and HFG as the major discriminants between the two groups being upregulated statistically significantly in group 2 (Figure S2D,E). Based on these analyses we marked the models in group 1 as low secretor cell lines and the models in group 2 as high secretor cell lines (Table 1).

### 3.3. The Cytokine Expression of CRC Cell Lines Was Significantly Influenced by the Culture Condition and the Presence of Fibroblasts

**Figure 3:**
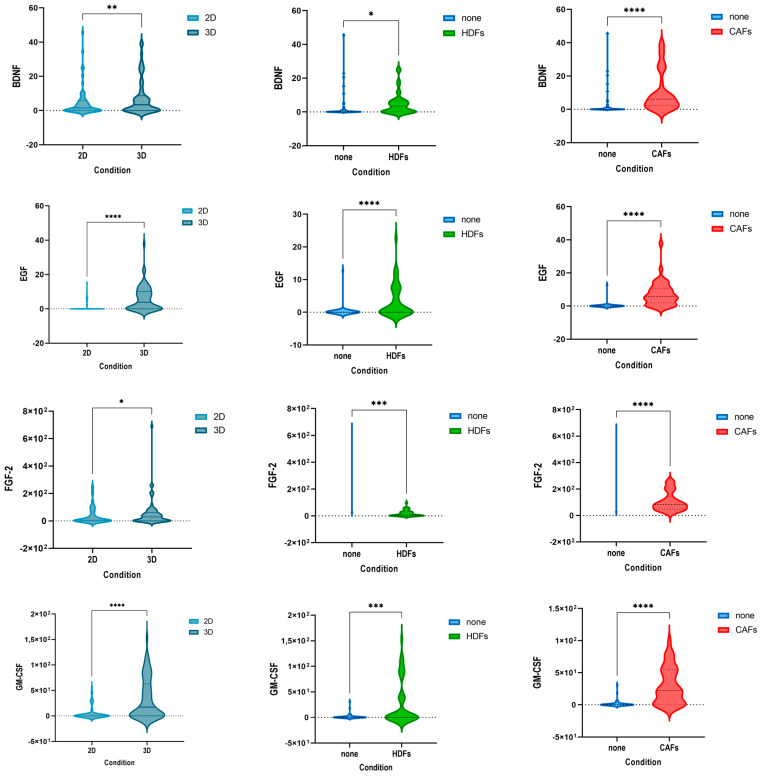

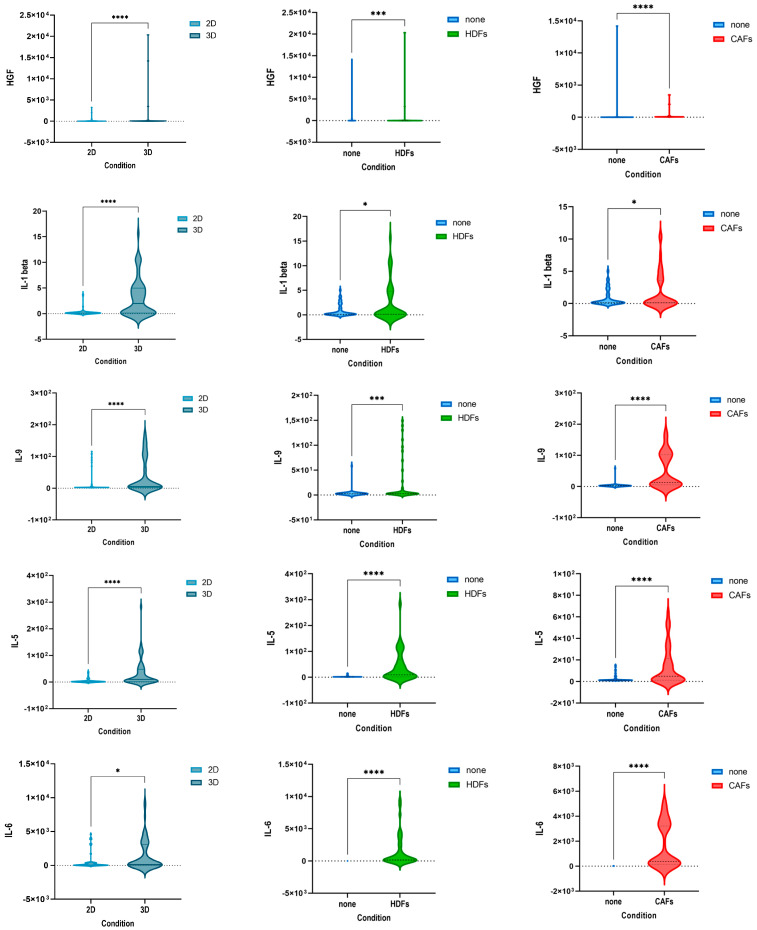

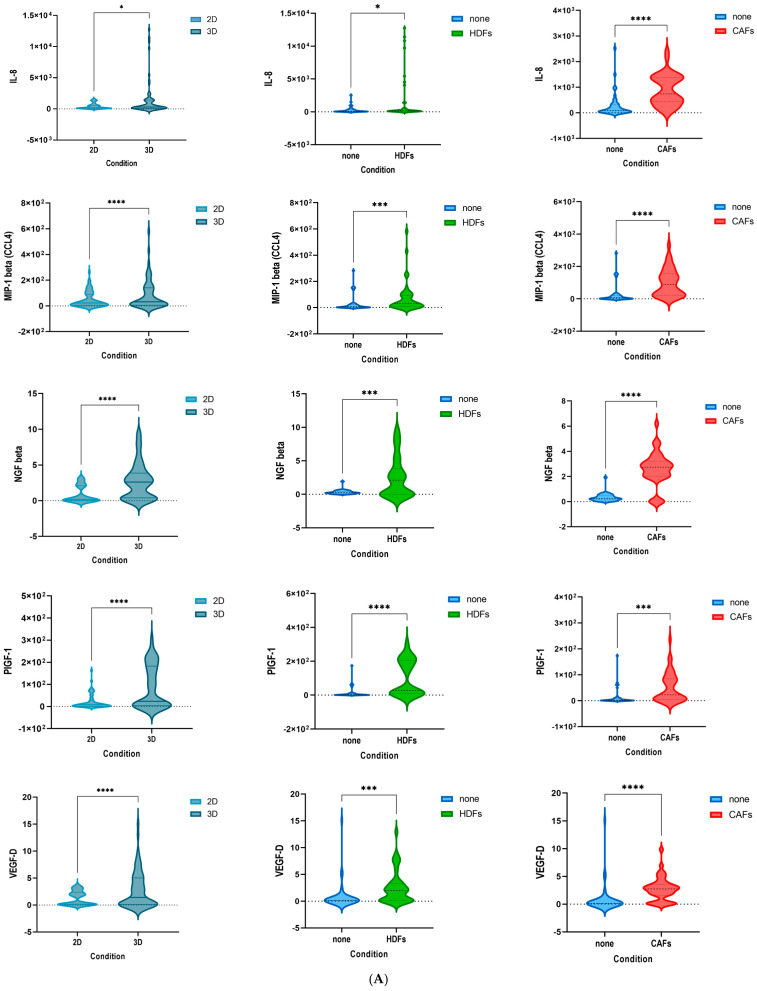

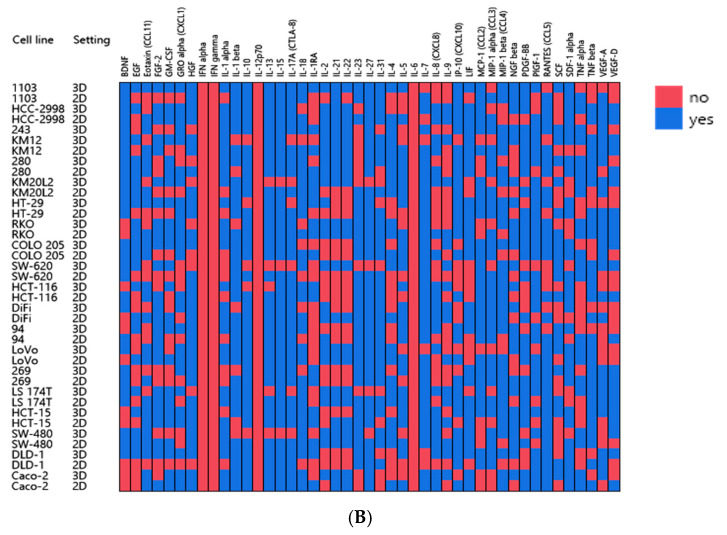
HDFs and CAFs similarly influence the secretome profiles of CRC cell lines, while cytokines and growth factors are statistically significant upregulated in 3D and co culture with fibroblasts. (**A**) Violin plots depicting cytokines which are statistically significantly upregulated in 3D and in coculture with HDFs and CAFs when compared to 2D and monoculture settings, respectively. *p*-values * < 0.05, ** < 0.01, *** < 0.001, **** < 0.0001 of Wilcoxon matched-pairs signed rank test are illustrated. (**B**) Cell plot of fold changes calculated for HDFs or CAFs vs. monoculture. “Yes” indicates that both HDFs and CAFs induce the same effect (up- or downregulation of the cytokine in the coculture condition with fibroblasts), whereas “no” shows the difference between the two types of fibroblasts: for 70% of the factors HDFs and CAFs equally influence the expression in the different conditions.

Of the secreted factors, 14 were statistically significantly upregulated in 3D vs. 2D and in coculture with HDFs and CAFs vs. monoculture (Figure 3A). Moreover, the differences of 26 other cytokines were statistically significant in the comparison 3D vs. 2D and/or coculture with one type of fibroblasts (HDFs and CAFs) vs. monoculture (Figure S3A,B). To summarize, a total of 40 out of 45 cytokines were statistically significantly upregulated in at least one analysis. A comparison between the cytokine modulation between both types of fibroblasts in 2D and 3D demonstrated that HDFs and CAFs had a similar effect on the cytokine profile: for 70% of the cytokines the change in expression was independent of the source of the fibroblasts (Figure 3B).

**Figure 4:**
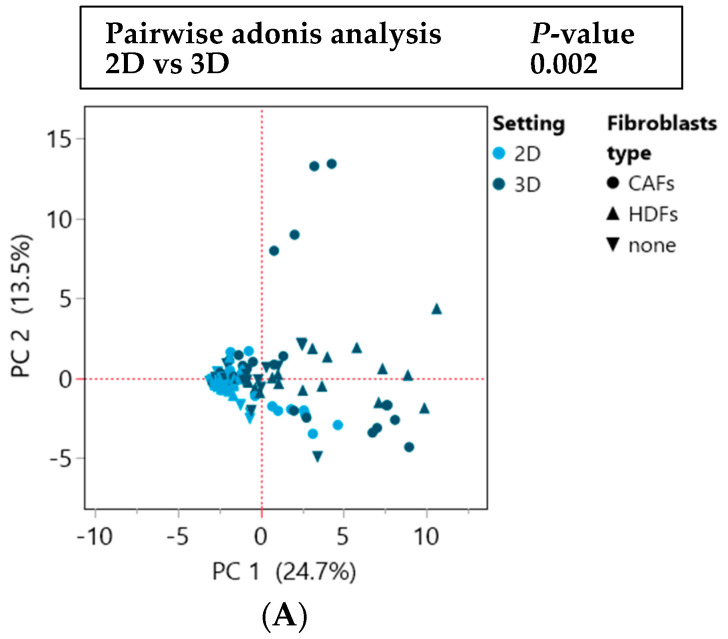

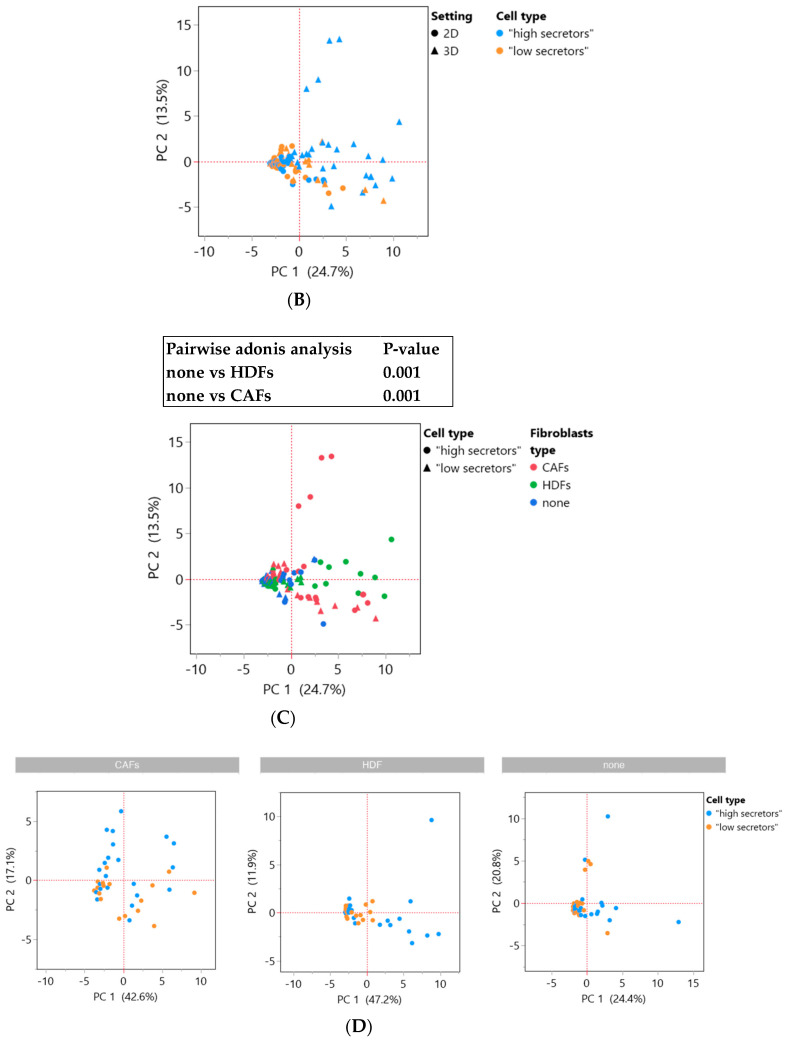

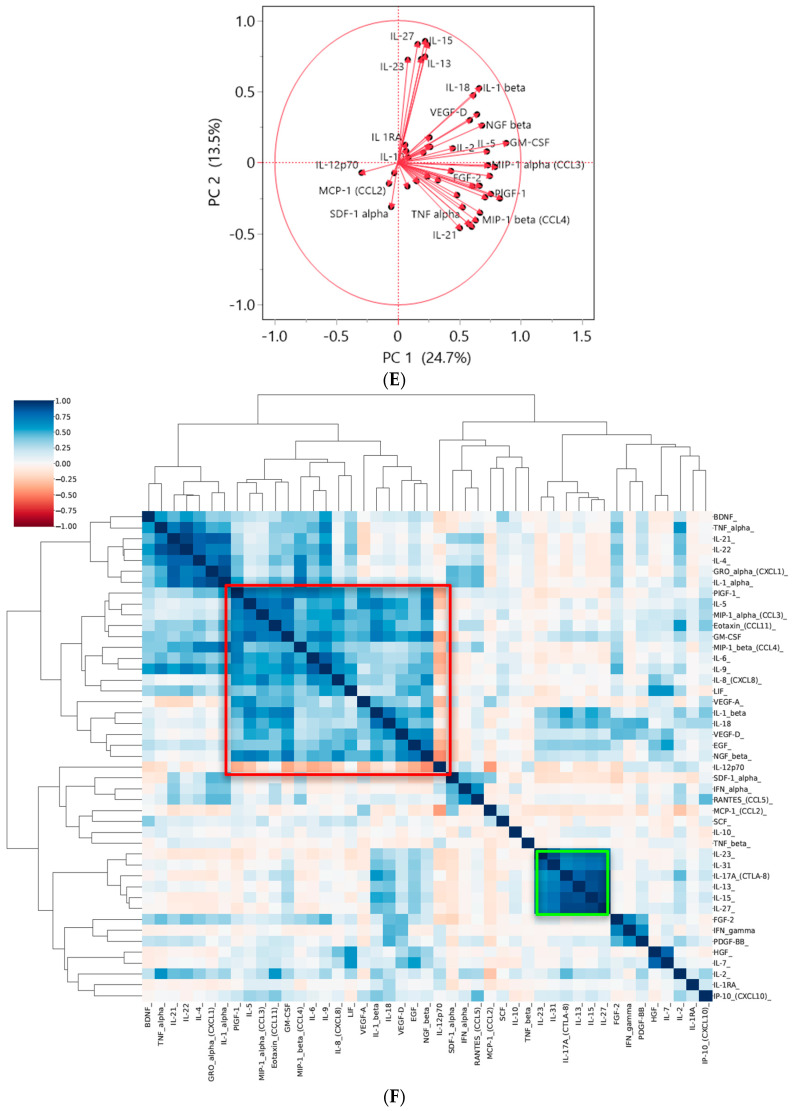
The separation of the cell lines into high and low secretors is more distinct in 3D than in 2D. (**A**–**C**) Principal component analysis (PCA) of cytokine profiles of 20 CRC cell lines in 2D and 3D settings in mono- and coculture with HDFs and CAFs. (**A**) and (**B**) plots highlight the differences between 2D and 3D settings and high and low secretor cell lines. (**C**) plot highlights the differences between the monoculture and coculture conditions and high and low secretor cell lines. (**D**) PCA analysis of secretome profiles of 20 CRC lines in 2D and 3D settings in the different mono- and coculture settings. (**E**) Loading plot of the PCA analysis of secretome profiles of 20 CRC cell lines in 2D and 3D settings in the different mono- and coculture conditions. (**F**) Pearson correlation coefficient between 2D and 3D of the secretome profiles of 20 CRC lines in mono- and coculture with HDFs and CAFs. Green and red squares highlight cytokines whose expressions are positively or negatively correlated between each other in the different settings. In the red square the differences of 14 out of the 16 cytokines are statistically significant between the 2D and 3D setting.

A principal component analysis (PCA) confirmed the observation from the cluster analysis: *p*-values of 0.002 for 2D vs. 3D and 0.0001 for mono- vs. coculture was displayed (Figure 4A–C). Moreover, a more diverse cytokine profile between the individual lines was observed in the 3D spheroid culture compared to the 2D setting regardless of the coculture condition (Figure 4A).

This effect was particularly evident for the tumor lines recognized as high secretor, whereas the low secretor cell lines grouped closer together regardless of the culture setting (Figure 4B). This was confirmed in the comparison between the monoculture and the coculture with fibroblasts, where the divergence between the individual CRC lines in the group of the high secretors in coculture with both types of fibroblasts was increased (Figure 4C,D). Of note, principal components 1 high vs. low secreting tumor lines were statistically significant in the comparisons 3D vs. 2D and coculture (HDFs) vs. monoculture (Figure S4B,E).

Two clusters of cytokines and growth factors were highly correlated in the 2D and 3D settings (green and red squares) (Figure 4F). In the green square, cytokines mainly expressed in the 3D setting or in coculture with CAFs paired together, while in the red square, cytokines mostly present in coculture with both types of fibroblasts were found. Of note, IL-12p70, which clustered with other cytokines in the red square, was negatively correlated with them, since it was only expressed in monoculture (Figure 4F). IL-12p70 showed to be inversely correlated with the other cytokines also in the PCA. Here, SDF-1 alpha and MCP-1 (CCL2) have the same trend as IL-12p70 (Figure 4E).

A partition analysis confirmed those observations, as SDF-1 alpha, MCP-1 (CCL2) and IL-12p70 were among the cytokines that differentiate at best the different settings (3D vs. 2D and coculture with HDFs and CAFs vs. monoculture) (Figure S4G,H,J).

### 3.4. The Investigated Tumor-Intrinsic Features of the CRC Lines Had No Major Impact on Their Cytokine Profile

**Figure 5:**
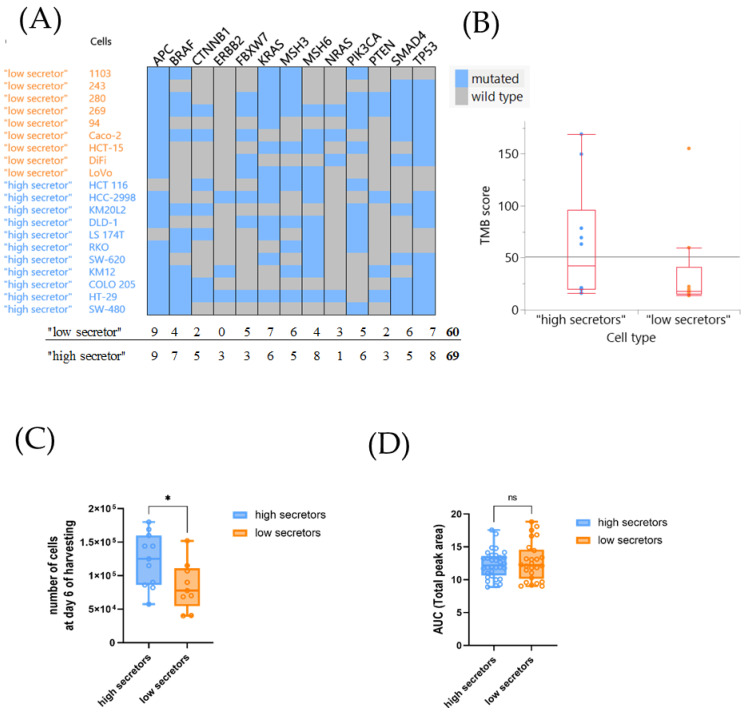
Comparison of tumor-intrinsic factors between the high and low secretor CRC tumor cell lines. (**A**) Mutational frequencies (WES data) of cancer specific genes of the CRC cell lines. (**B**) Kruskal–Wallis test (rank sums) between the TMB scores of the two groups. (**C**) Calculated number of cells present at day 6 in the 2D culture setting of the two groups of cell lines based on the doubling times of each cell line. (**D**) Proliferation rate of the cancer cells in the 3D spheroids culture setting. AUC of the largest brightfield object areas (µm^2^) of the spheroids in monoculture for both groups are shown. (**C**,**D**) Error bars represent SEM, *p*-values * <0.05, ^ns^ non significant of Mann–Whitney test (nonparametric comparison test) are illustrated.

To understand the tumor-intrinsic influence on the cytokine profile we evaluated the tumor mutational burden (TMB), the presence of driver mutations and the proliferation rate. The molecular phenotype of the CRC lines indicated higher mutational frequencies of cancer specific genes (Figure 5A, 69 vs. 60), and higher TMB (mean: 43.63 vs. 25.07) scores in the high compared to the low secreting cell lines. However, when analyzing the CRC specific mutation panel, a clear pattern between the two groups based on specific genetic mutations was not evident (Figure 5A). Furthermore, the difference in the TMB scores was not statically significant (Figure 5B, Figure S5A).

In parallel, we assessed a correlation between the proliferation rates of the CRC lines and the cytokine profiles. For the 2D setting, an estimation of cell growth was performed by calculating the number of cells at day 6, i.e., the day the supernatant was harvested for the cytokine expression analysis, based on the doubling times of each cell line (see M&M) (Figure S5B). For the 3D spheroid culture setting, the AUC for each cell line was calculated as an indicator of spheroid growth in the 3D setting (Figure S5C). While both groups grew similarly as spheroids in the 3D setting, a low statistically significant difference between the two groups was identified in 2D (Figure 5C,D). When analyzing the available metadata of the donor patients a trend towards primary tumor as the source of the cells was observed for high secretor lines. In contrast, most of the low secreting tumor lines were derived from metastatic lesions. This observation has to be taken with caution as the data set was very small and incomplete, and the donor-patient metadata did not indicate the actual disease status. The patients from the high secretor group were younger than the one from the low secretor group (46 years vs. 56 years). Of note, the data for age as well as for sex were incomplete, decreasing the statistical power of the analysis (Table S1).

### 3.5. The Co-Culture with Different Tumor Cell Lines and Fibroblasts Influence the Macrophage Polarization Distinctively

To analyze the cross-talk between macrophages, tumor cells and other parts of the TME, we cultured macrophages together with three different tumor cell lines (HCT-116, HT-29 both high secretors and 269, low secretor) with either HDFs or CAFs in 3D. In parallel to the cytokine analysis described below, the polarization of the macrophages was examined by FC to determine how the cross-talk among the different cells modulated the phenotype of the immune cells.

**Figure 6:**
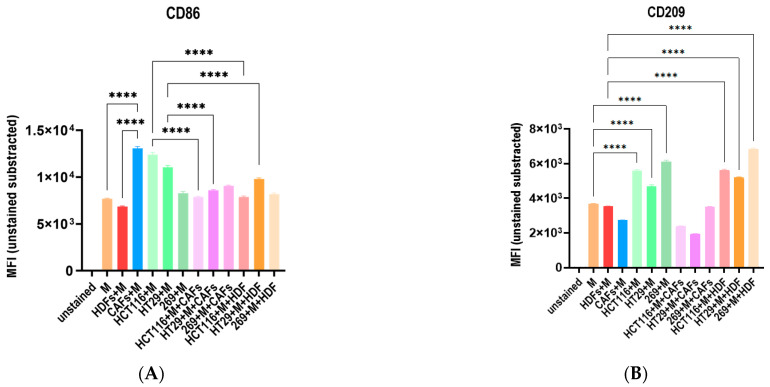

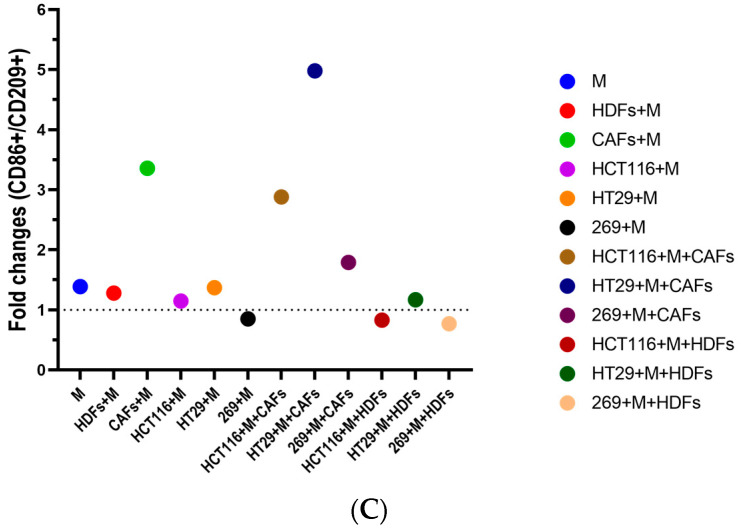
Determination of macrophage phenotype in 3D. 1 × 10⁴ colon cancer cell lines (HCT-116, HT-29 and 269) and 6 × 10^3^ of two fibroblast lines, human dermal fibroblasts (HDFs) or cancer-associated fibroblasts (CAFs) were cultured together in ultra-low adhesion (ULA) round-bottom 96-well plates for 2 days. On day 3, 5 × 10⁴ macrophages from healthy donors were added to the wells as mono-, co- and triple cultures after resuspension in a matrigel–collagen matrix. After 6 days the polarization of the macrophages was examined by flow cytometry (FC) based on the expression of CD45, CD11b, CD86 and CD209. (**A**,**B**) CD86 and CD209 MFIs are indicated in the bar graph. MFI of unstained sample was subtracted from the other samples. n = 10; M = Macrophages; CAFs = Cancer associated fibroblasts; HDFs = Human dermal fibroblasts. Error bars represent SEM, *p*-values **** < 0.0001 of one-way ANOVA (Holm–Šídák multiple comparison test) are illustrated. (**C**) Fold changes of the CD86+/CD209+ mean fluorescence intensities (MFI) are illustrated.

CD86 as representative M1 marker was upregulated in coculture with CAFs and the two high secretor tumor cell lines HCT-116 and HT-29. The coculture with HDFs as well as all triple culture conditions did not impact the expression level of CD86 on the macrophages (Figure 6A). CD209 as representative M2 marker was upregulated in coculture with all three tumor cell lines and in triple culture with HDFs (Figure 6B). As a consequence, the coculture with CAFs increased the M1/M2 ratio. In the triple culture with CAFs and the individual tumor cell lines the high secretors increased the M1/M2 ratio, whereas the low secretor cell line 296 shifted the ratio towards an M2 phenotype. HDFs did not influence the expression of CD86 or CD209 on macrophages in any of the tested culture conditions (Figure 6C).

### 3.6. The Cocultures with Macrophages and Fibroblasts Modulates the Cytike Profile and Confirms the Impact of the Fibrobast Source on Macrophage Polarization

**Figure 7:**
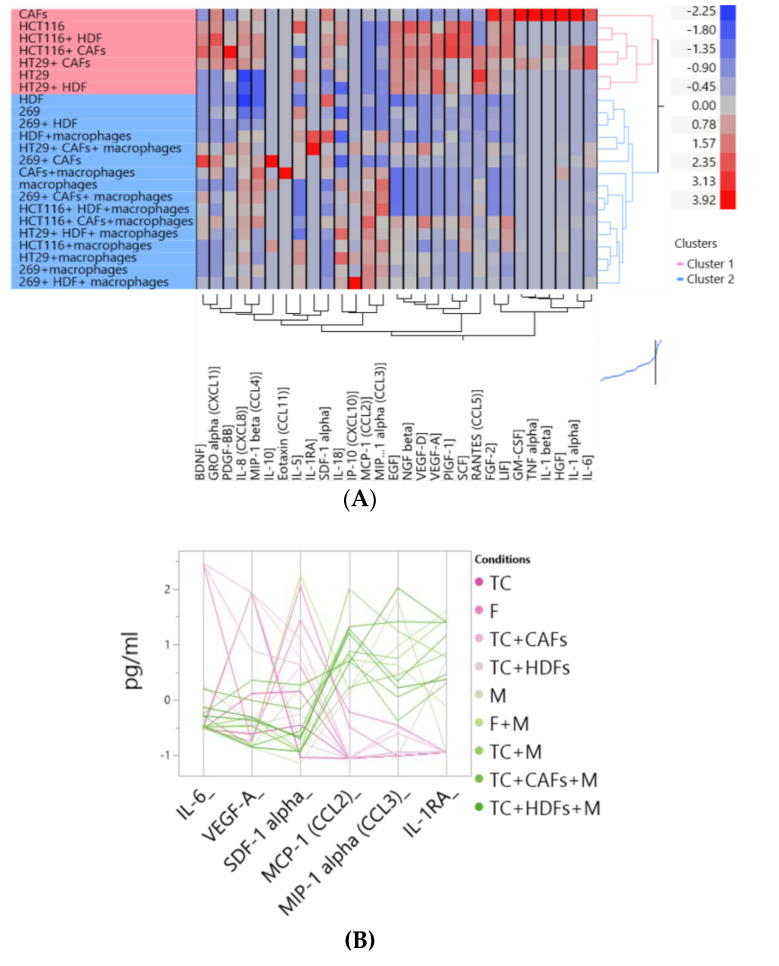

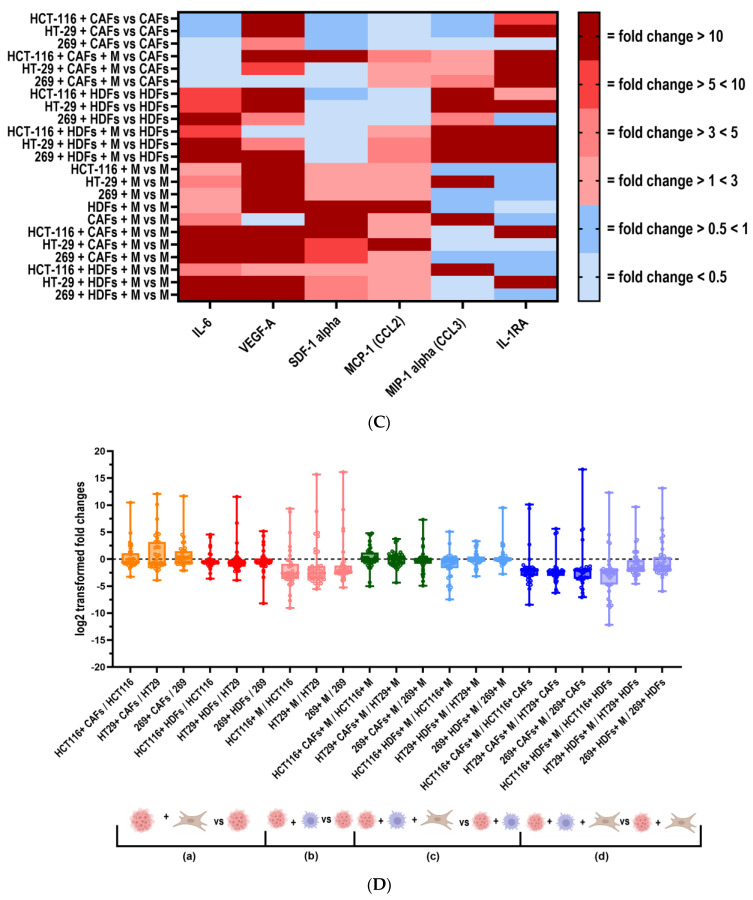
(**A**) Cytokine profiles of CRC cell lines in coculture with fibroblasts and macrophages in 3D. Supernatants from mono-, co- and triple cultures were collected on day 12 and the expression of 45 cytokines was determined with a human-specific magnetic bead-based assay. Cytokine levels data (pg/mL) are illustrated in the heatmap. Before clustering, the values were standardized. Hierarchical clustering (Method = Ward) is shown. n = 2. (**B**) Parallel plot of IL-6, SDF-1 alpha, VEGF-A, MCP-1, MIP-1 alpha, IL-1RA levels data (pg/mL) in mono-, co- and triple cultures. TC = triple culture; F = fibroblasts; M = macrophages. (**C**) MCP-1 (CCL2), MIP-1 alpha (CCL3), IL-1RA, VEGF-A; SDF-1 alpha, IL-6 fold changes are displayed in the heatmap. M = Macrophages; CAFs = Cancer associated fibroblasts; HDFs = Human dermal fibroblasts. (**D**) Log2 transformed fold changes (a) tumor cells in coculture with CAFs (orange) or HDFs (red) vs. tumor cells in monoculture; (b) tumor cells in coculture with macrophages vstumor cells in monoculture (pink); (c) tumor cells in triple culture with macrophages and CAFs (green) or HDFs (light blue) vs. tumor cells in coculture with macrophages; (d) tumor cells in triple culture with macrophages and CAFs (dark blue) or HDFs (violet) vs. tumor cells in coculture with CAFs (dark blue) or HDFs (violet).

A cluster analysis of the cytokine data defined two major clusters mainly separated by the presence or absence of macrophages (Figure 7A). A subset of cytokines displayed a specific profile between the different culture conditions: the addition of macrophages to any coculture induced an increase of MCP-1 (CCL2), MIP-1 alpha (CCL3) and IL-1Rα in the supernatant, while the presence of both types of fibroblasts induced an enhanced secretion of VEGF-A, SDF-1 alpha and for CAFs IL-6. VEGF-A and SDF-1 were highly secreted by all tumor lines in mono- and coculture with fibroblasts, while their secretion was downregulated in the presence of macrophages (Figure 7B). The comparison of the fold changes between the different settings highlighted the influence of the different cell types on the expression of specific cytokines. Compared to macrophages in monoculture, the secretion of IL-6, VEGF-A, SDF-1 and MCP-1 was increased in the presence of other cell types, whereas the secretion of MIP-1 and IL-1RA was decreased. The addition of CAFs induced an increase of CCL3 whereas HDFs caused an increase of CCL2, which is in line with their specific influence on the M1/M2 ratio (Figure 7C). Across all cytokines and coculture conditions the addition of macrophages reduced the amount of secreted factors independent of the tumor cell line or fibroblast source (Figure 7D).

## 4. Discussion

The cancer cytokine profile represents a valuable source of biomarkers, and therefore holds promise to be exploited to improve diagnosis, prognosis and disease monitoring of CRC [18,19].

Our approach to better characterize the CRC cytokine profile in vitro comprised the analysis of 20 CRC lines representing different subtypes of the disease in the presence or absence of fibroblasts and macrophages in 2D and 3D [20,21].

This study displayed an upregulation of cytokine levels when the cell lines were cultured as tumor spheroids in 3D. Spheroids are one of the most used 3D cell models due to their ability to retain tumor features [22]. Hence, the overexpression of factors involved in inflammation, EMT and angiogenesis in the 3D spheroid culture, shown by this analysis, highlights the necessity of accurately mimicking the three dimensional orientation of tumor cells in situ. The coculture with fibroblasts induced an overexpression of cytokines involved in inflammation, angiogenesis and cell invasion. Similar effects were observed when colon cancer cells were cultured as monoculture in 3D.

A number of angiogenic, pro-inflammatory and EMT related-factors were exclusively expressed in 3D or in coculture with HDFs and CAFs. This finding supports the importance of the TME by functionally stimulating a dysregulated secretion and all the following malignant processes, which contribute to tumorigenesis and cancer progression [6].

Of note, this analysis highlighted that HDFs and CAFs had an analogous influence on the cytokine profile, since the overexpression of the majority of secreted factors was mutually induced by both types of fibroblasts.

The overexpression of secreted factors observed in the presence of HDFs and not only of CAFs suggested an activation of the fibroblasts driven by the cancer cells. Indeed, the aberrant production of TGF-β1, osteopontin, interleukin-1β by cancer cells is considered one of the principal mechanisms through which normal fibroblasts differentiate to CAFs [23,24,25]. Thus, in our analysis, the overexpression of these factors could be caused by the cancer cells, triggered by the presence of HDFs or by the HDFs themselves, that are transformed into CAFs when they come into contact with the tumor cells [26].

Based on the cluster analysis of the cytokine profiles the CRC lines were separated into two groups. One group was annotated as low secretors and the other as high secretors.

LIF and HGF were recognized as the main discriminant between those groups. LIF plays a tumor-suppressive role in CRC by activating STAT3 pathway and downregulating p53 protein level [27]. Although its tumorigenic function in CRC is still poorly understood, high levels of LIF in CRC are associated with chemoresistance and poor prognosis [27,28]. Given the highly significant difference in the expression levels of high and low secretor lines, we sustain the importance of further exploring the tumorigenic functions of LIF for potential future therapeutic applications in CRC. HGF is well described as an inducer of resistance towards targeted therapy, pro-tumorigenic and pro-metastatic factor in the TME [26,29,30].

In this study VEGF-A and IL-6 levels were increased particularly in coculture with fibroblasts and in the presence of extracellular matrix. IL-6 secretion by fibroblasts seemed to be activated by the cancer cells under inflammatory and hypoxic conditions [31,32]. IL-6 can activate STAT3 pathway and enhance VEGF production, thereby promoting CRC cell growth and angiogenesis [31].

On the contrary, IL-12p70 (a pro-inflammatory cytokine that promotes antitumor T helper type 1 responses) confirmed its suitability to be used as a potential prognostic and predictive biomarker for CRC [33]. Our results displayed a unique secretion of IL-12p70 in monoculture across the CRC cell line panel. The secretion was downregulated with increasing complexity of the culture system, which went hand in hand with an increase in tumor proliferation and a pro-angiogenic, pro-inflammatory cytokine profile. The TME of CRC suppresses IL-12p70 secretion, which is also considered a predictive marker for resistance towards anti-angiogenic therapy [33,34]. Although a prospective clinical trial has yet to prove that hypothesis, our findings strengthen the argument in favor of IL-12p70 as a prognostic and predictive marker for CRC.

The addition of macrophages to any coculture increased IL-1Rα, CCL2 and CCL3 in the supernatant. High secretion of IL-1Rα induces EMT and cell proliferation. CCL2 and CCL3 influence monocyte recruitment and production of MMP9, thus promoting tumor invasion and metastasis [35,36,37]. The translational relevance of this finding was proven by Chun et al., who described how the antibody-mediated neutralization or inhibition of CCL2 can prevent the myeloid cell recruitment mediated by CCL2 and arrest cancer progression [38]. CCL2 is specifically produced by macrophages during inflammation and released by CAFs–macrophage cocultures. On the contrary CAFs do to not release this chemokine alone [11,39]. In line with the literature, high levels of CCL2 were detected specifically when macrophages were present.

In our model the different types of fibroblasts and tumor cells influence the macrophage differentiation in a very distinct way. The increase in the M1/M2 ratio specifically seen with CAFs was supported by the increase of CCL3 [40]. Vice versa, the boost towards M2 by HDFs was confirmed by the increased secretion of CCL2 in the relevant conditions [41]. Since tumor-associated macrophages mainly accumulate in the stroma and rarely infiltrate the tumor epithelium, their recruitment and differentiation is recognized as directly dependent on signals from stromal cells [42]. Likewise, we identified that CAFs influenced macrophage differentiation at most, while only the addition of one of the three cell lines, HT-29, boosted polarization in the triple culture with CAFs. This reinforces the pivotal role of CAFs in influencing macrophage differentiation.

## 5. Conclusions

Taken together, we have thoroughly characterized the cytokine profile of 20 CRC lines recapitulating the heterogeneity of the disease in different in vitro culture conditions including two types of fibroblasts, HDFs and CAFs, as well as macrophages, as a major representative of the TME.

Our data proved a significant influence of the TME on tumor biology, especially exerted on tumor lines identified as high secretors by cluster analysis. The differences highlighted between the recognized high and low secretor cell lines in terms of cytokine profile and influence exerted by the TME proved to be strictly linked to specific cytokines related to angiogenesis, EMT and invasion. Furthermore, individual key cytokines were identified and their importance as promising biomarker candidates for CRC discussed.

## Figures and Tables

**Table 1:** Table of the clusters obtained from the three heatmaps: 18 cell lines belong to the same cluster in the 3D monoculture (Figure 2A) and 3D coculture with HDFs (Figure 2B) divisions; 16 of them also belong to the same cluster also in the fold changes heatmap (Figure 2C).

Cell Lines																					
		94	243	269	280	1103	Caco-2	DiFi	HCT-15	LoVo	COLO 205	DLD-1	HCC-2998	HCT 116	HT-29	KM12	KM20L2	LS 174T	RKO	SW-480	SW-620
cluster	3D monoculture	1	1	1	1	1	1	1	1	1	1	2	3	1	2	2	2	2	4	2	2
	3D co-culture	1	1	1	1	1	1	1	1	1	2	2	2	2	2	2	2	2	2	1	1
	3D mono- vs. co-culture fold change	1	3	3	3	1	1	1	3	3	1	1	2	1	1	2	1	2	2	3	2
group		1	1	1	1	1	1	1	1	1	2	2	2	2	2	2	2	2	2	2	2
		low secretor									high secretor										

## Data Availability

The data presented in this study are available within the article [and/or its Supplementary Materials].

## Supplementary Materials

The following supporting information can be downloaded at: https://www.mdpi.com/article/10.3390/cells12212539/s1, Figure S1: Calculation of fold changes between 3D and 2D settings and between coculture with fibroblasts and monoculture for each condition; Figure S2: Cytokine profile profiles of 20 CRC cell lines in 3D spheroid cultures and in coculture with CAFs; Figure S3: Cytokines levels in different settings and cell lines types; Figure S4: The cytokine profile of the “high-secretor” lines is positively influenced by the 3D culture setting and the presence of fibroblasts; Figure S5: TMB scores and proliferation rate of the CRC cell lines; Table S1: Metadata about CRC cell lines: name, 2D and 3D assay doubling times, type of cell line based on cluster analysis, patient histology, gender, patient age at surgery, pathogenic status, site of origin.

## References

[1] Pretzsch E., Bösch F., Neumann J., Ganschow P., Bazhin A., Guba M., Werner J., Angele M. (2019). Mechanisms of Metastasis in Colorectal Cancer and Metastatic Organotropism: Hematogenous versus Peritoneal Spread. J. Oncol..

[2] Farhan H., Rabouille C. (2011). Signalling to and from the secretory pathway. J. Cell Sci..

[3] La Vecchia S., Sebastián C. (2020). Metabolic pathways regulating colorectal cancer initiation and progression. Semin. Cell Dev. Biol..

[4] Karagiannis G.S., Pavlou M.P., Diamandis E.P. (2010). Cancer secretomics reveal pathophysiological pathways in cancer molecular oncology. Mol. Oncol..

[5] Maggio M.C., Miniaci A., Gallizzi R., Civino A. (2023). “Neuroimmunoendocrinology” in Children with Rheumatic Diseases: How Glucocorticoids Are the Orchestra Director. Int. J. Mol. Sci..

[6] da Cunha B.R., Domingos C., Stefanini A.C.B., Henrique T., Polachini G.M., Castelo-Branco P., Tajara E.H. (2019). Cellular Interactions in the Tumor Microenvironment: The Role of Secretome. J. Cancer.

[7] Javarsiani M.H., Javanmard S.H., Colonna F. (2019). Metastatic components in colorectal cancer. J. Res. Med. Sci..

[8] Rudisch A., Dewhurst M.R., Horga L.G., Kramer N., Harrer N., Dong M., van der Kuip H., Wernitznig A., Bernthaler A., Dolznig H. (2015). High EMT Signature Score of Invasive Non-Small Cell Lung Cancer (NSCLC) Cells Correlates with NFκB Driven Colony-Stimulating Factor 2 (CSF2/GM-CSF) Secretion by Neighboring Stromal Fibroblasts. PLoS ONE.

[9] Alkasalias T., Moyano-Galceran L., Arsenian-Henriksson M., Lehti K. (2018). Fibroblasts in the Tumor Microenvironment: Shield or Spear?. Int. J. Mol. Sci..

[10] Deng L., Jiang N., Zeng J., Wang Y., Cui H. (2021). The Versatile Roles of Cancer-Associated Fibroblasts in Colorectal Cancer and Therapeutic Implications. Front. Cell Dev. Biol..

[11] Fujimoto H., Sangai T., Ishii G., Ikehara A., Nagashima T., Miyazaki M., Ochiai A. (2009). Stromal MCP-1 in mammary tumors induces tumor-associated macrophage infiltration and contributes to tumor progression. Int. J. Cancer.

[12] Lin Y., Xu J., Lan H. (2019). Tumor-associated macrophages in tumor metastasis: Biological roles and clinical therapeutic applications. J. Hematol. Oncol..

[13] Cho H., Seo Y., Loke K.M., Kim S.-W., Oh S.-M., Kim J.-H., Soh J., Kim H.S., Lee H., Kim J. (2018). Cancer-Stimulated CAFs Enhance Monocyte Differentiation and Protumoral TAM Activation via IL6 and GM-CSF Secretion. Clin. Cancer Res..

[14] Pinto M.L., Rios E., Durães C., Ribeiro R., Machado J.C., Mantovani A., Barbosa M.A., Carneiro F., Oliveira M.J. (2019). The Two Faces of Tumor-Associated Macrophages and Their Clinical Significance in Colorectal Cancer. Front. Immunol..

[15] Schneider M., Huber J., Hadaschik B., Siegers G.M., Fiebig H.H., Schüler J. (2012). Characterization of colon cancer cells: A functional approach characterizing CD133 as a potential stem cell marker. BMC Cancer.

[16] Néron S., Thibault L., Dussault N., Côté G., Ducas É., Pineault N., Roy A. (2007). Characterization of mononuclear cells remaining in the leukoreduction system chambers of apheresis instruments after routine platelet collection: A new source of viable human blood cells. Transfusion.

[17] Brown W.E., Hu J.C., Athanasiou K.A. (2016). Ammonium-Chloride-Potassium Lysing Buffer Treatment of Fully Differentiated Cells Increases Cell Purity and Resulting Neotissue Functional Properties. Tissue Eng. Part C Methods.

[18] Tjalsma H., Bolhuis A., Jongbloed J.D., Bron S., van Dijl J.M. (2000). Signal peptide-dependent protein transport in Bacillus subtilis: A genome-based survey of the secretome. Microbiol. Mol. Biol. Rev..

[19] Cevenini A., Orrù S., Imperlini E. (2020). Secretome Proteomic Approaches for Biomarker Discovery: An Update on Colorectal Cancer. Medicina.

[20] Chen F., Zhuang X., Lin L., Yu P., Wang Y., Shi Y., Hu G., Sun Y. (2015). New horizons in tumor microenvironment biology: Challenges and opportunities. BMC Med..

[21] Xing F., Saidou J., Watabe K. (2010). Cancer associated fibroblasts (CAFs) in tumor microenvironment. Front. Biosci..

[22] Rodrigues J., Heinrich M.A., Teixeira L.M., Prakash J. (2021). 3D In Vitro Model (R)evolution: Unveiling Tumor-Stroma Interactions. Trends Cancer.

[23] LeBleu V.S., Kalluri R. (2018). A peek into cancer-associated fibroblasts: Origins, functions and translational impact. Dis. Models Mech..

[24] Toti A., Santi A., Pardella E., Nesi I., Tomasini R., Mello T., Paoli P., Caselli A., Cirri P. (2021). Activated fibroblasts enhance cancer cell migration by microvesicles-mediated transfer of Galectin-1. J. Cell Commun. Signal..

[25] Harper J., Sainson R.C. (2014). Regulation of the anti-tumour immune response by cancer-associated fibroblasts. Semin. Cancer Biol..

[26] Dias Carvalho P., Mendonça S., Martins F., Oliveira M.J., Velho S. (2022). Modulation of Fibroblast Phenotype by Colorectal Cancer Cell-Secreted Factors Is Mostly Independent of Oncogenic KRAS. Cells.

[27] Yu H., Yue X., Zhao Y., Li X., Wu L., Zhang C., Liu Z., Lin K., Xu-Monette Z.Y., Young K.H. (2014). LIF negatively regulates tumour-suppressor p53 through Stat3/ID1/MDM2 in colorectal cancers. Nat. Commun..

[28] Zhang C., Liu J., Wang J., Hu W., Feng Z. (2021). The emerging role of leukemia inhibitory factor in cancer and therapy. Pharmacol. Ther..

[29] Takumi Y., Arai S., Suzuki C., Fukuda K., Nishiyama A., Takeuchi S., Sato H., Matsumoto K., Sugio K., Yano S. (2023). MET kinase inhibitor reverses resistance to entrectinib induced by hepatocyte growth factor in tumors with NTRK1 or ROS1 rearrangements. Cancer Med..

[30] Xiong M., Wang M., Yan Y., Chen X., Guo W., Xu M., Guo S., Wang Y. (2022). MACC1 Promotes the Progression and Is a Novel Biomarker for Predicting Immunotherapy Response in Colorectal Cancer. J. Oncol..

[31] Grivennikov S., Karin E., Terzic J., Mucida D., Yu G.Y., Vallabhapurapu S., Scheller J., Rose-John S., Cheroutre H., Eckmann L. (2009). IL-6 and Stat3 are required for survival of intestinal epithelial cells and development of colitis-associated cancer. Cancer Cell.

[32] Nagasaki T., Hara M., Nakanishi H., Takahashi H., Sato M., Takeyama H. (2014). Interleukin-6 released by colon cancer-associated fibroblasts is critical for tumour angiogenesis: Anti-interleukin-6 receptor antibody suppressed angiogenesis and inhibited tumour-stroma interaction. Br. J. Cancer.

[33] Michielsen A.J., Ryan E.J., O’Sullivan J.N. (2012). Dendritic cell inhibition correlates with survival of colorectal cancer patients on bevacizumab treatment. Oncoimmunology.

[34] O’Toole A., Michielsen A.J., Nolan B., Tosetto M., Sheahan K., Mulcahy H.E., Winter D.C., Hyland J.M., O’Connell P.R., Fennelly D. (2014). Tumour microenvironment of both early- and late-stage colorectal cancer is equally immunosuppressive. Br. J. Cancer.

[35] Chen Y., Yang Z., Deng B., Wu D., Quan Y., Min Z. (2020). Interleukin 1β/1RA axis in colorectal cancer regulates tumor invasion, proliferation and apoptosis via autophagy. Oncol. Rep..

[36] Li J., Huang L., Zhao H., Yan Y., Lu J. (2020). The Role of Interleukins in Colorectal Cancer. Int. J. Biol. Sci..

[37] Li Y., Lei Y., Sun J., Zhang W., Li X., Chen S., Kong D., Chen C., Bi K., Luo X. (2022). A promising research direction for colorectal cancer immunotherapy: The regulatory mechanism of CCL5 in colorectal cancer. Front. Oncol..

[38] Chun E., Lavoie S., Michaud M., Gallini C.A., Kim J., Soucy G., Odze R., Glickman J.N., Garrett W.S. (2015). CCL2 Promotes Colorectal Carcinogenesis by Enhancing Polymorphonuclear Myeloid-Derived Suppressor Cell Population and Function. Cell Rep..

[39] Stadler M., Pudelko K., Biermeier A., Walterskirchen N., Gaigneaux A., Weindorfer C., Harrer N., Klett H., Hengstschläger M., Schüler J. (2021). Stromal fibroblasts shape the myeloid phenotype in normal colon and colorectal cancer and induce CD163 and CCL2 expression in macrophages. Cancer Lett..

[40] Yuan X., Liu W., Li Y., Chen K., Li H., Tang H., Yin Y., Song Z., Chen D. (2023). CCL3 aggravates intestinal damage in NEC by promoting macrophage chemotaxis and M1 macrophage polarization. Pediatr. Res..

[41] Pan B., Wan T., Zhou Y., Huang S., Yuan L., Jiang Y., Zheng X., Liu P., Xiang H., Ju M. (2023). The MYBL2-CCL2 axis promotes tumor progression and resistance to anti-PD-1 therapy in ovarian cancer by inducing immunosuppressive macrophages. Cancer Cell Int..

[42] Strasser K., Birnleitner H., Beer A., Pils D., Gerner M.C., Schmetterer K.G., Bachleitner-Hofmann T., Stift A., Bergmann M., Oehler R. (2019). Immunological differences between colorectal cancer and normal mucosa uncover a prognostically relevant immune cell profile. Oncoimmunology.

