# Supplementary material for: A Subset of Colon Cancer Cell Lines Displays a Cytokine Profile Linked to Angiogenesis, EMT and Invasion Which Is Modulated by the Culture Conditions In Vitro
Source: Cells. 2023 Oct 29;12(21):2539. doi: 10.3390/cells12212539 (PMC10648033; doi:10.3390/cells12212539)
Supplement: Supplementary file 1 [file cells-12-02539-s001.zip › cells-2659340-supplementary.pdf]

## Supplementary material

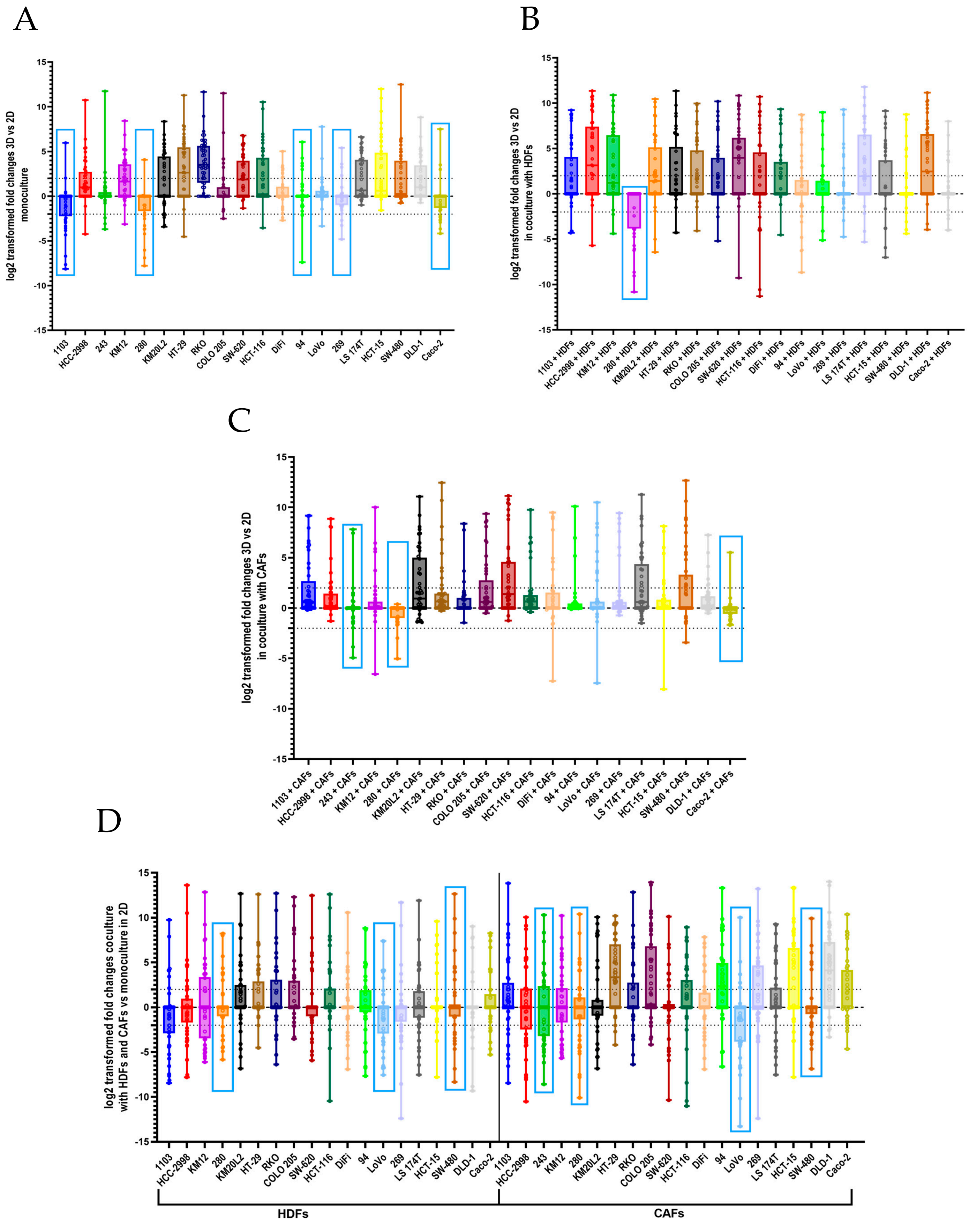

E

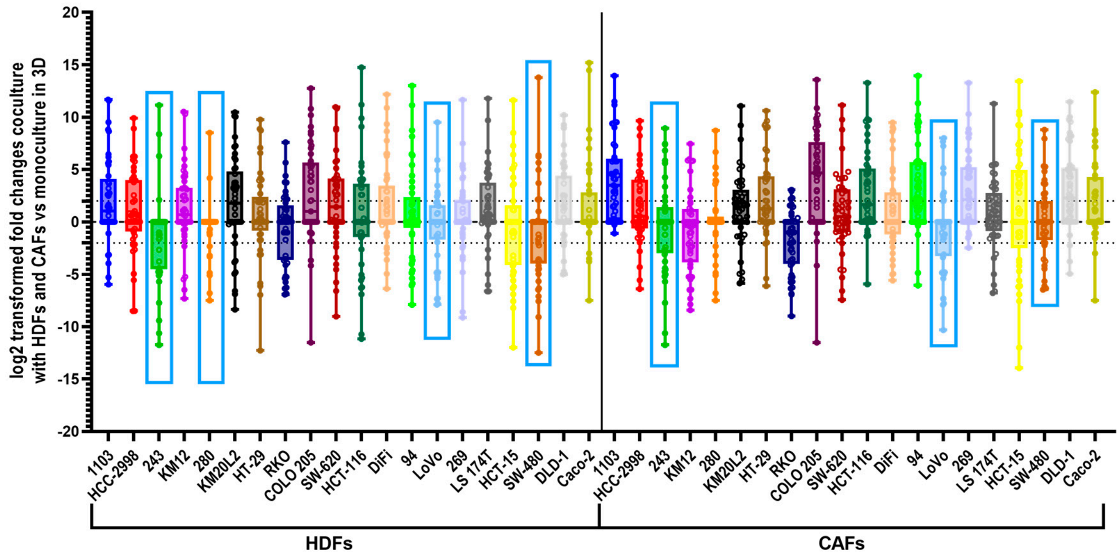


**Figure S1. Calculation of fold changes between 3D and 2D settings and between coculture with fibroblasts and monoculture for each condition. A.** Log2 transformed fold changes 3D vs 2D monoculture **B.** Log2 transformed fold changes 3D vs 2D coculture with HDFs. **C.** Log2 transformed fold changes 3D vs 2D coculture with CAFs. **D.** Log2 transformed fold changes coculture with HDFs and CAFs vs monoculture in 2D. E. Log2 transformed fold changes coculture with HDFs and CAFs vs monoculture in 3D.

A

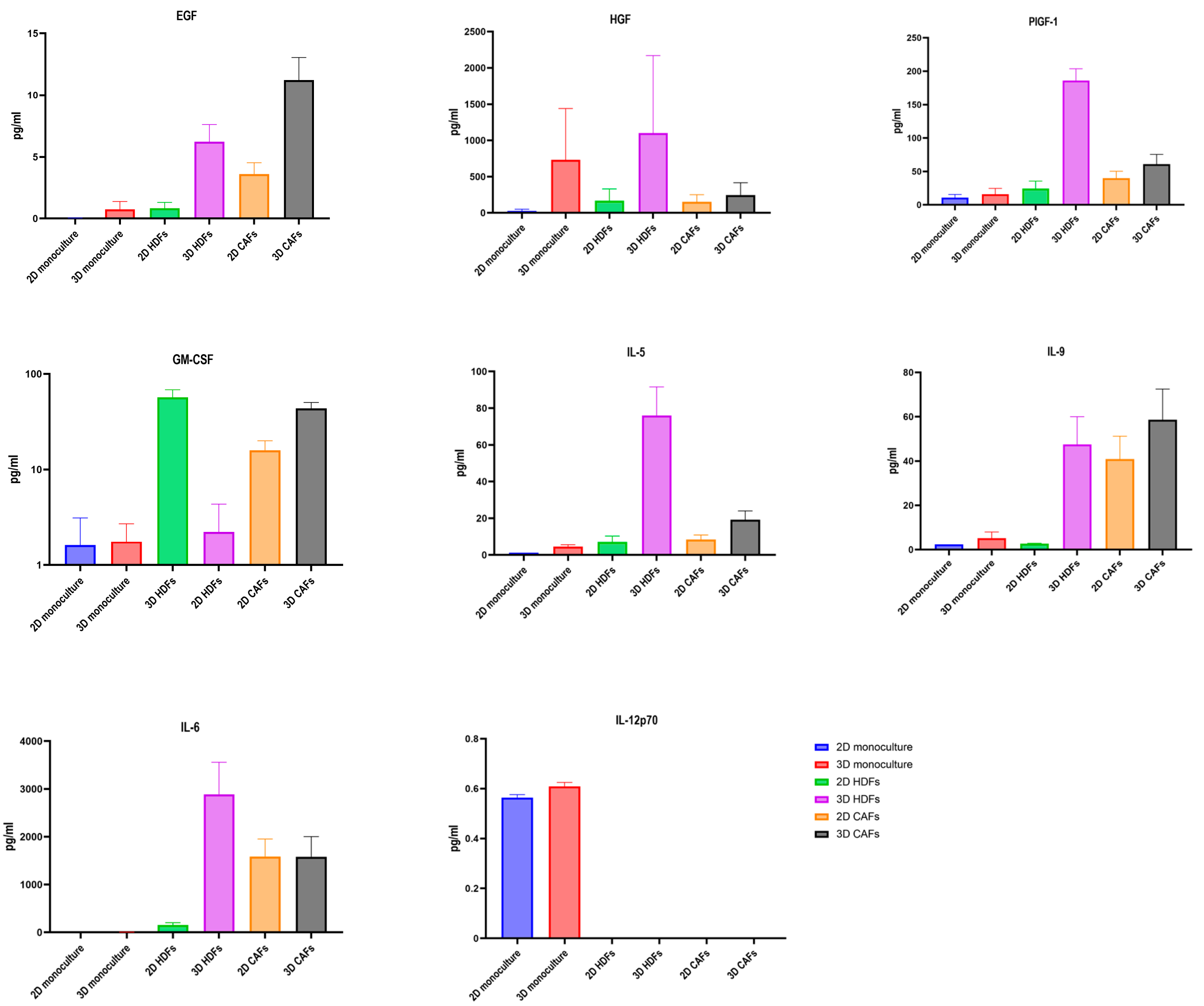
EGF
pg/ml
0
5
10
15
2D monoculture
3D monoculture
2D HDFs
3D HDFs
2D CAFs
3D CAFs
HGF
pg/ml
0
500
1000
1500
2000
2500
PlGF-1
pg/ml
0
50
100
150
200
250
GM-CSF
pg/ml
1
10
100
IL-5
pg/ml
0
20
40
60
80
100
IL-9
pg/ml
0
20
40
60
80
IL-6
pg/ml
0
1000
2000
3000
4000
IL-12p70
pg/ml
0
0.2
0.4
0.6
0.8
2D monoculture
3D monoculture
2D HDFs
3D HDFs
2D CAFs
3D CAFs

B

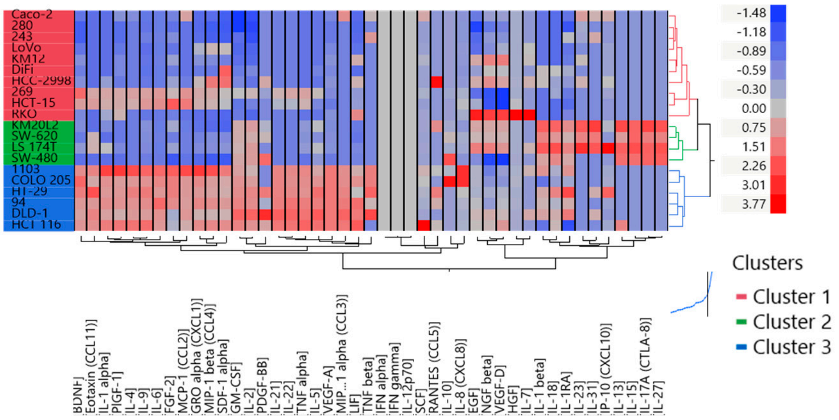

Caco-2
280
243
LoVo
KM12
DiFi
HCC-2998
269
HCT-15
RKO
KM20L2
SW-620
LS 174T
SW-480
1103
COLO 205
HT-29
94
DLD-1
HCT 116
-1.48
-1.18
-0.89
-0.59
-0.30
0.00
0.75
1.51
2.26
3.01
3.77
Clusters
Cluster 1
Cluster 2
Cluster 3


C

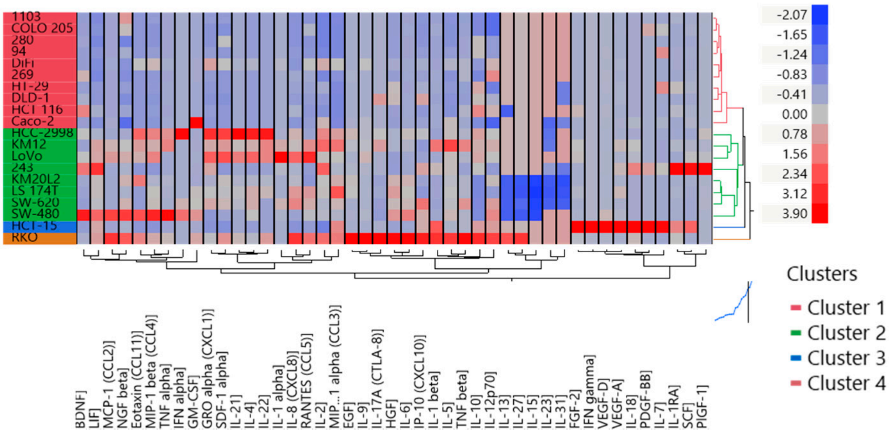

1103
COLO 205
280
94
DiFi
269
HT-29
DLD-1
HCT 116
Caco-2
HCC-2998
KM12
LoVo
243
KM20L2
LS 174T
SW-620
SW-480
HCT-15
RKO
-2.07
-1.65
-1.24
-0.83
-0.41
0.00
0.78
1.56
2.34
3.12
3.90
Clusters
Cluster 1
Cluster 2
Cluster 3
Cluster 4


D

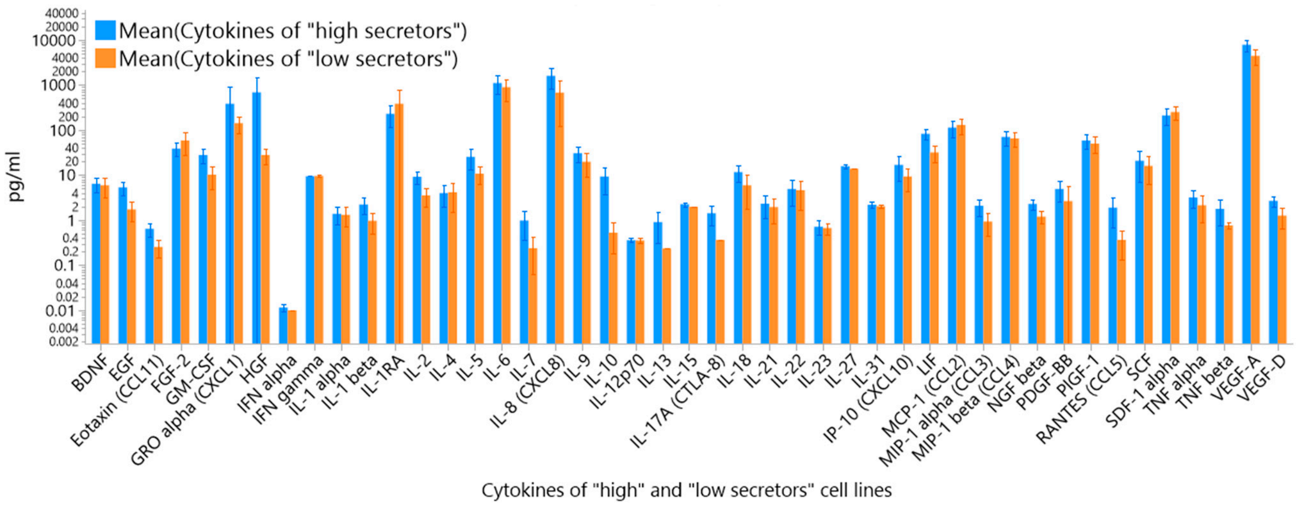

Mean(Cytokines of "high secretors")
Mean(Cytokines of "low secretors")
pg/ml
BDNF
EGF
Eotaxin (CCL11)
FGF-2
GM-CSF
GRO alpha (CXCL1)
HGF
IFN alpha
IFN gamma
IL-1 alpha
IL-1 beta
IL-1RA
IL-2
IL-4
IL-5
IL-6
IL-7
IL-8 (CXCL8)
IL-9
IL-10
IL-12p70
IL-13
IL-15
IL-17A (CTLA-8)
IL-18
IL-21
IL-22
IL-23
IL-27
IL-31
IP-10 (CXCL10)
LIF
MCP-1 (CCL2)
MIP-1 alpha (CCL3)
MIP-1 beta (CCL4)
NGF beta
PDGF-BB
PIGF-1
RANTES (CCL5)
SCF
SDF-1 alpha
TNF alpha
TNF beta
VEGF-A
VEGF-D
Cytokines of "high" and "low secretors" cell lines

E

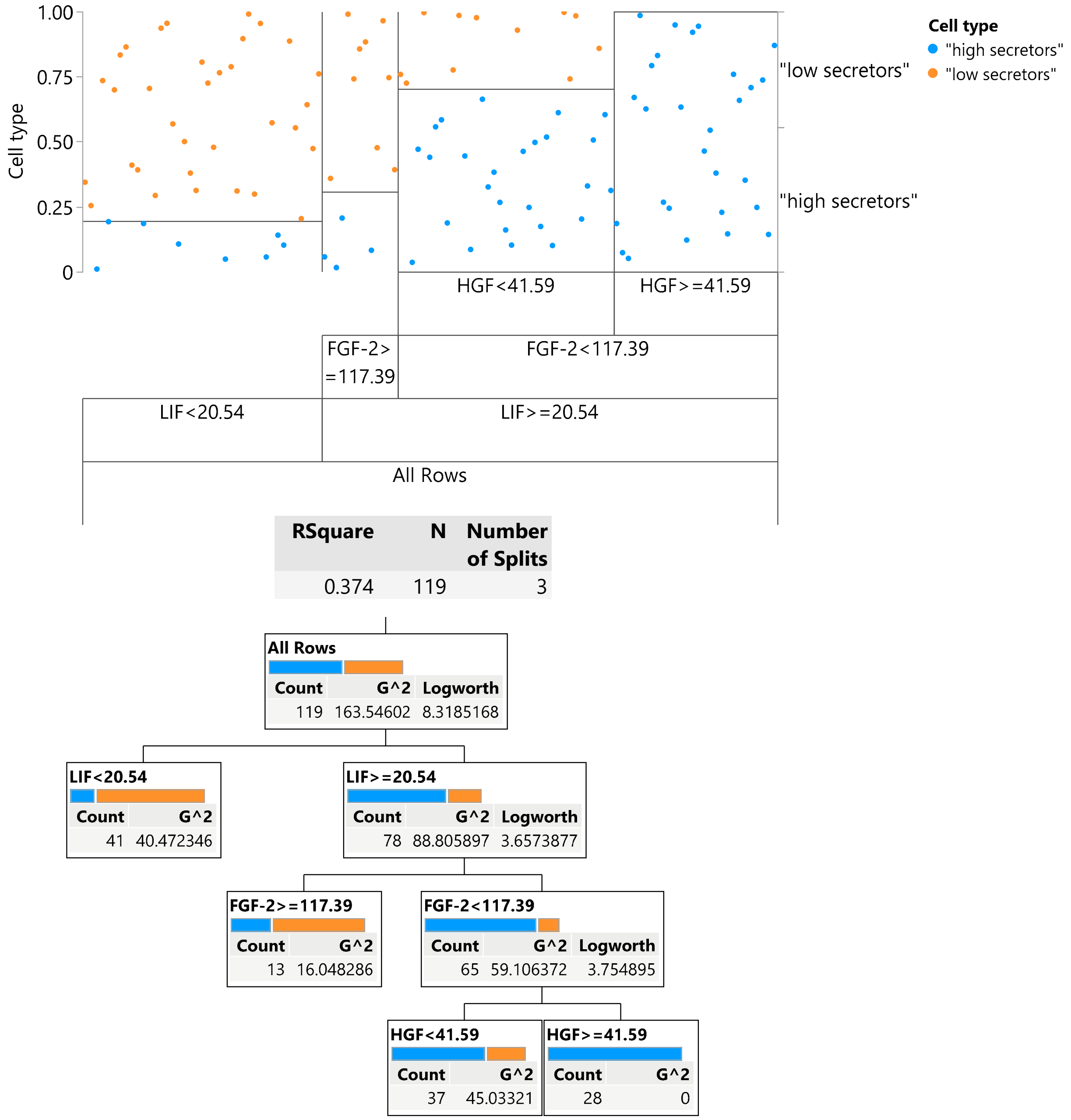


**Figure S2. Cytokine profiles of 20 CRC cell lines in 3D spheroid cultures and in coculture with CAFs. A.** GM-CSF, EGF, HGF, IL-5, IL-9, IL-6, IL-12p70, PIGF-1 pg/ml values are displayed in the bar graphs. **B.** Heat map of the cytokines levels (pg/ml) in 3D spheroids coculture with CAFs. **C.** Heat map of the fold changes coculture with CAFs vs 3D monoculture. Before clustering, B heatmaps values were standardized with values from Fig.2 A and B, while C heatmap values were standardized separately with values of Fig. 2 C. Hierarchical clustering (Method = Ward) is shown. n=2. **D.** Levels data (pg/ml) of cytokines in 2D and 3D in monoculture and coculture with HDFs and CAFs of "high and low secretor" CRC cell lines. Error bars represent Confidence intervals, Kruskal-Wallis test (Rank Sums) was carried out. P-values illustrating statistically significant difference can be found in Statistical analysis Fig. S2.pdf. Secreted factors are listed by goodness of fit. **E.** Partition plots and decision trees show the cytokines that explain at the most the differences between the "high and low secretor" cell lines.

A

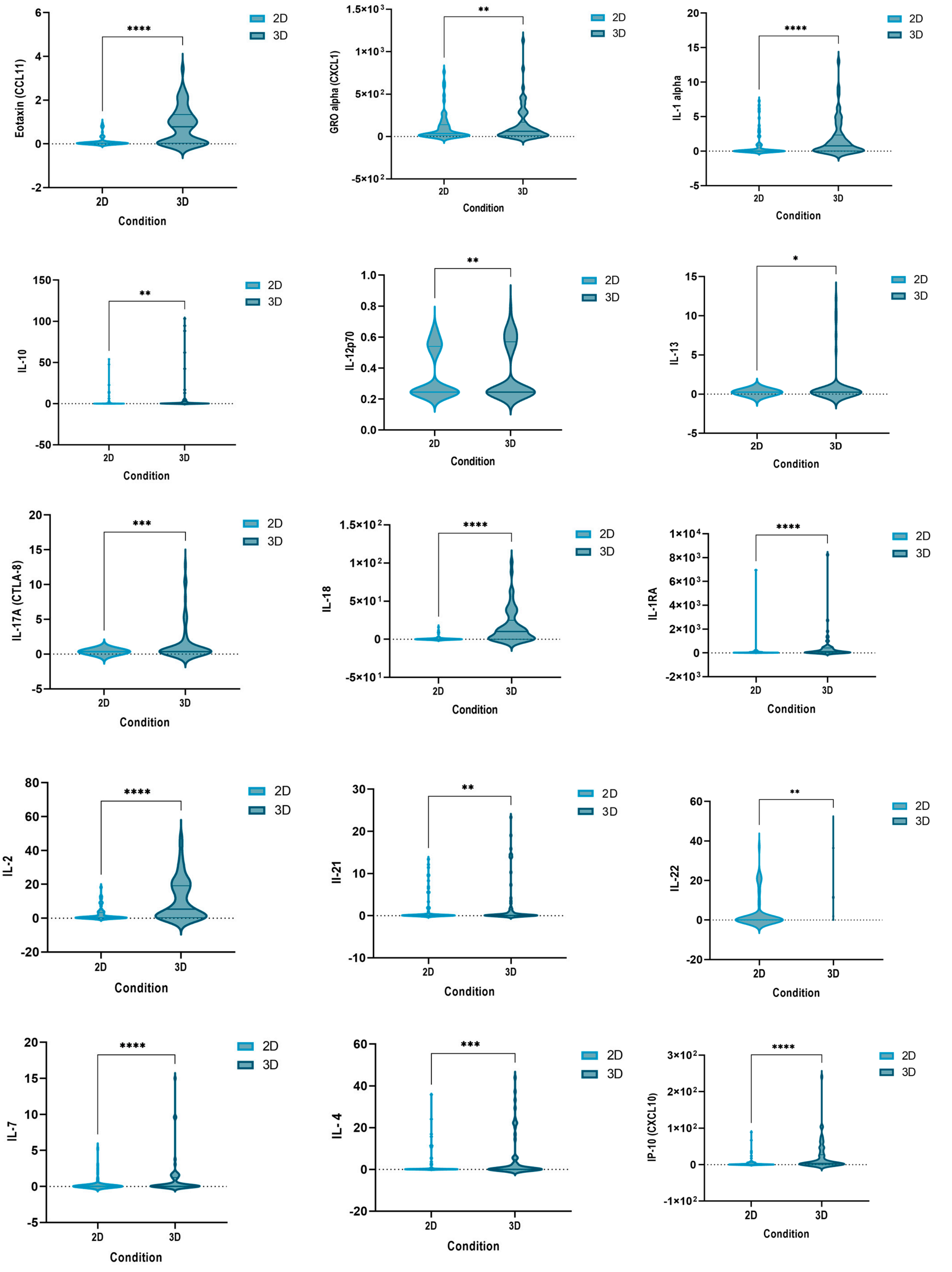

Eotaxin (CCL11)
6
4
2
0
-2
****
2D
3D
Condition
GRO alpha (CXCL1)
1.5×10³
1×10³
5×10²
0
-5×10²
**
IL-1 alpha
20
15
10
5
0
-5
****
IL-10
150
100
50
0
-50
**
IL-12p70
1.0
0.8
0.6
0.4
0.2
0.0
**
IL-13
15
10
5
0
-5
*
IL-17A (CTLA-8)
20
15
10
5
0
-5
***
IL-18
1.5×10²
1×10²
5×10¹
0
-5×10¹
****
IL-1RA
1×10⁴
8×10³
6×10³
4×10³
2×10³
0
-2×10³
****
IL-2
80
60
40
20
0
-20
****
Il-21
30
20
10
0
-10
**
IL-22
60
40
20
0
-20
**
IL-7
20
15
10
5
0
-5
****
IL- 4
60
40
20
0
-20
***
IP-10 (CXCL10)
3×10²
2×10²
1×10²
0
-1×10²
****

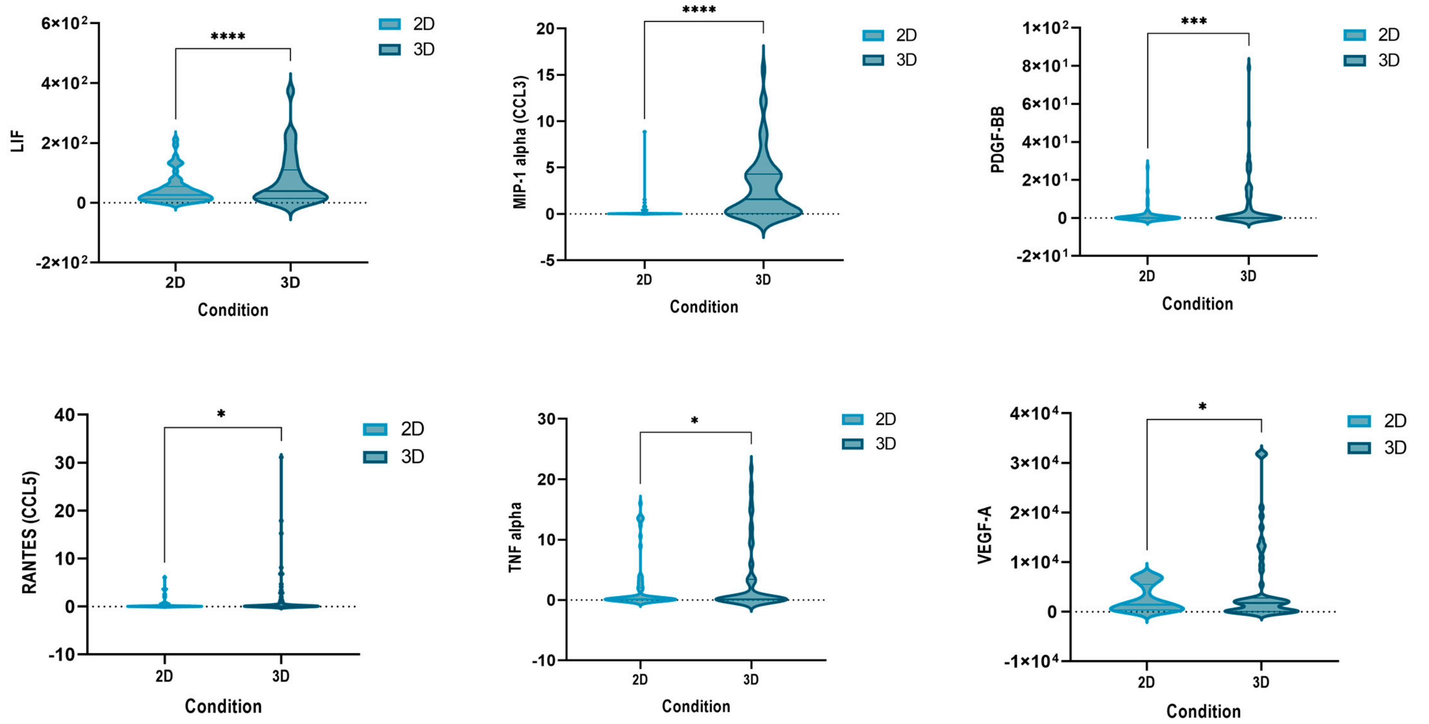
LIF
6×10²
4×10²
2×10²
0
-2×10²
****
2D
3D
Condition
MIP-1 alpha (CCL3)
20
15
10
5
0
-5
****
Condition
PDGF-BB
1×10²
8×10¹
6×10¹
4×10¹
2×10¹
0
-2×10¹
***
Condition
RANTES (CCL5)
40
30
20
10
0
-10
*
Condition
TNF alpha
30
20
10
0
-10
*
Condition
VEGF-A
4×10⁴
3×10⁴
2×10⁴
1×10⁴
0
-1×10⁴
*
Condition

B

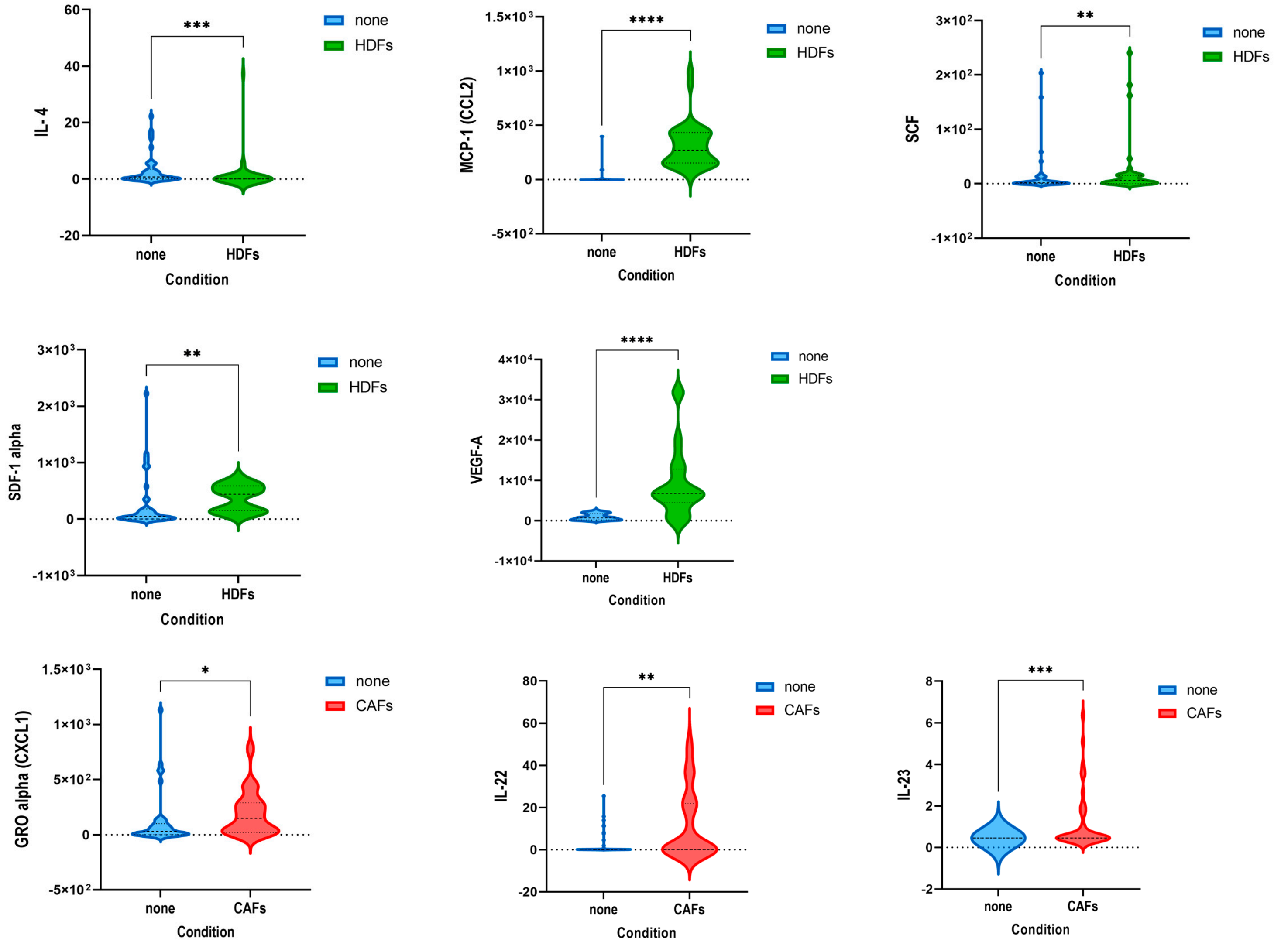
IL-4
60
40
20
0
-20
***
none
HDFs
Condition
MCP-1 (CCL2)
1.5×10³
1×10³
5×10²
0
-5×10²
****
Condition
SCF
3×10²
2×10²
1×10²
0
-1×10²
**
Condition
SDF-1 alpha
3×10³
2×10³
1×10³
0
-1×10³
**
Condition
VEGF-A
4×10⁴
3×10⁴
2×10⁴
1×10⁴
0
-1×10⁴
****
Condition
GRO alpha (CXCL1)
1.5×10³
1×10³
5×10²
0
-5×10²
*
none
CAFs
Condition
IL-22
80
60
40
20
0
-20
**
Condition
IL-23
8
6
4
2
0
-2
***
Condition

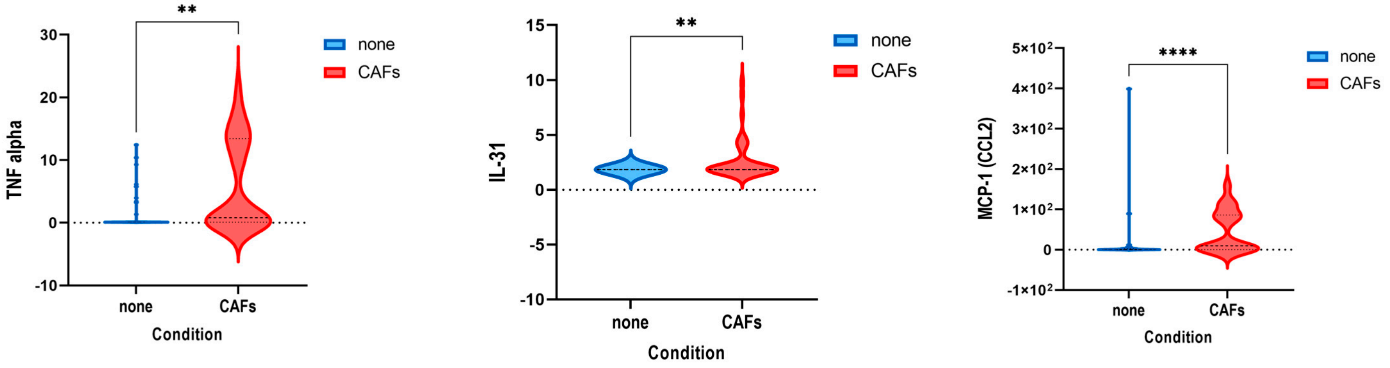


**Figure S3. Cytokines levels in different settings and cell lines types. A.** Violin plots depicting cytokines statistically significant upregulated in 3D when compared to 2D setting. **B.** Violin plots depicting cytokines statistically significant upregulated in coculture with HDFs and/or CAFs when compared to monoculture setting. P-values *< 0.05, **< 0.01, ***< 0.001, **** < 0.0001 of Wilcoxon matched-pairs signed rank test are illustrated.

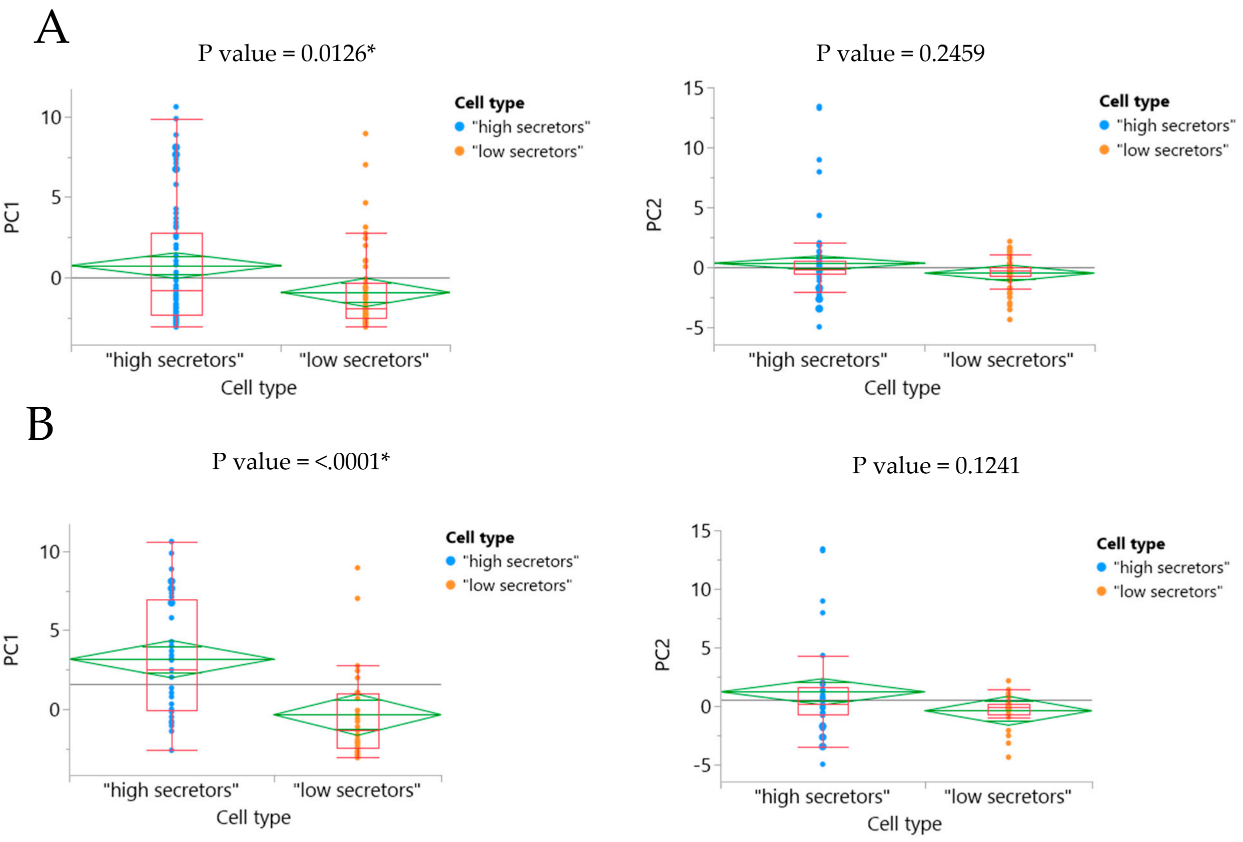

C

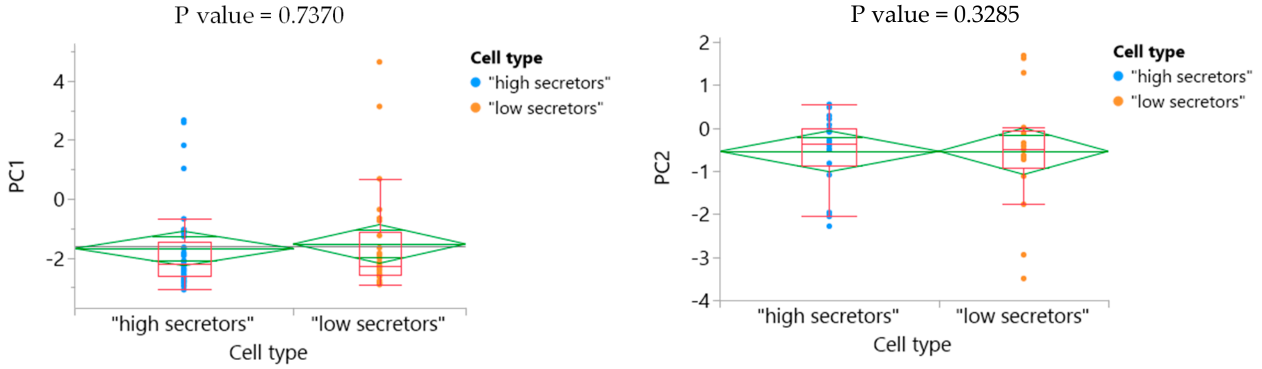
P value = 0.7370
PC1
Cell type
"high secretors"
"low secretors"
P value = 0.3285
PC2

D

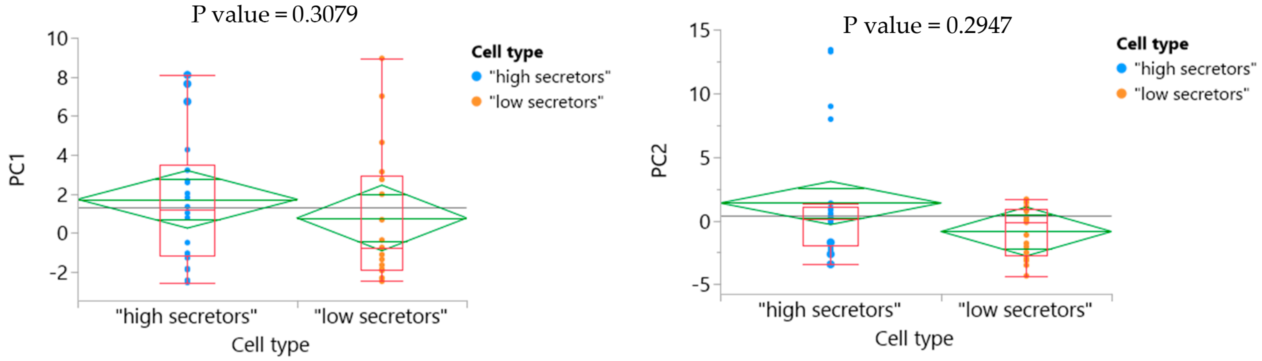
P value = 0.3079
PC1
Cell type
"high secretors"
"low secretors"
P value = 0.2947
PC2

E

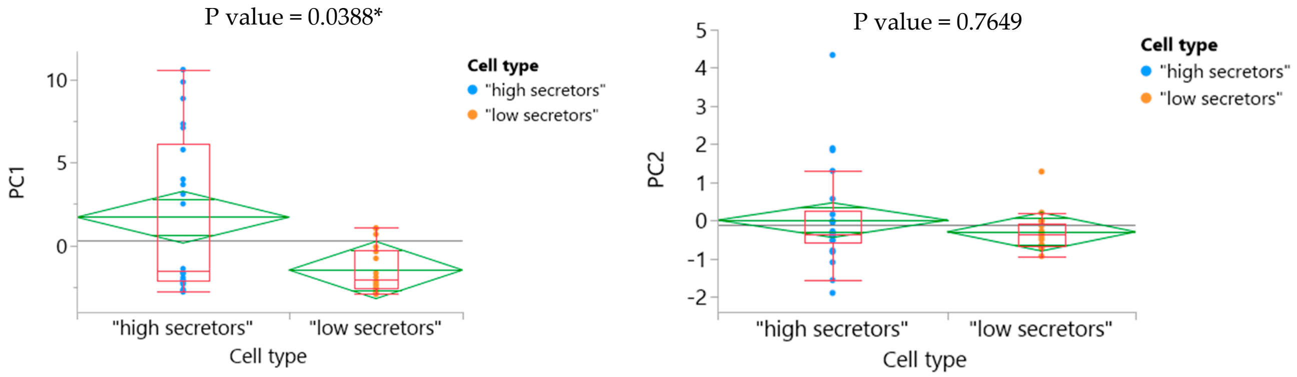
P value = 0.0388*
PC1
Cell type
"high secretors"
"low secretors"
P value = 0.7649
PC2

F

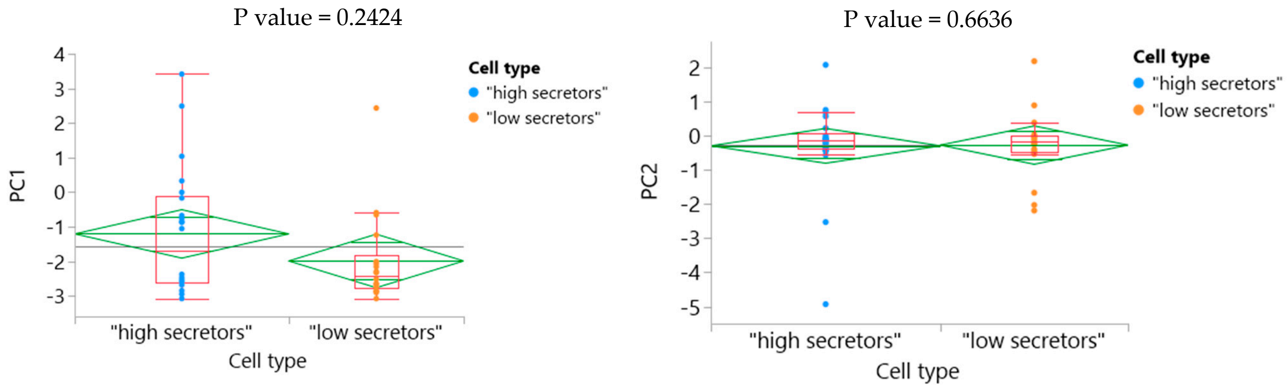
P value = 0.2424
PC1
Cell type
"high secretors"
"low secretors"
P value = 0.6636
PC2

G

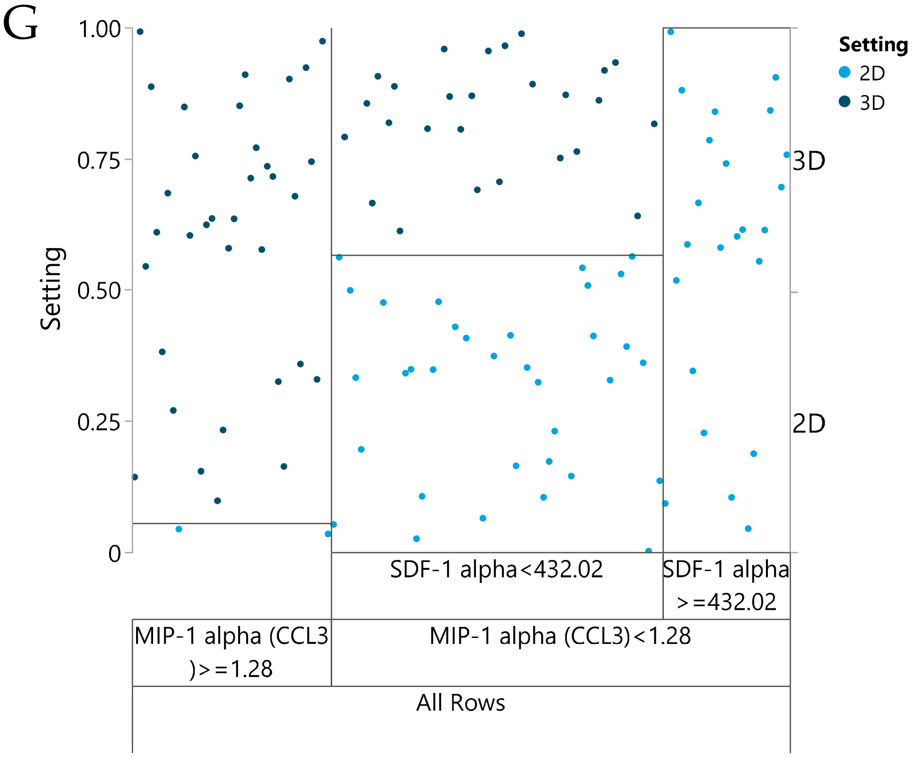


| RSquare | N | Number of Splits |
|---|---|---|
| 0.402 | 119 | 2 |

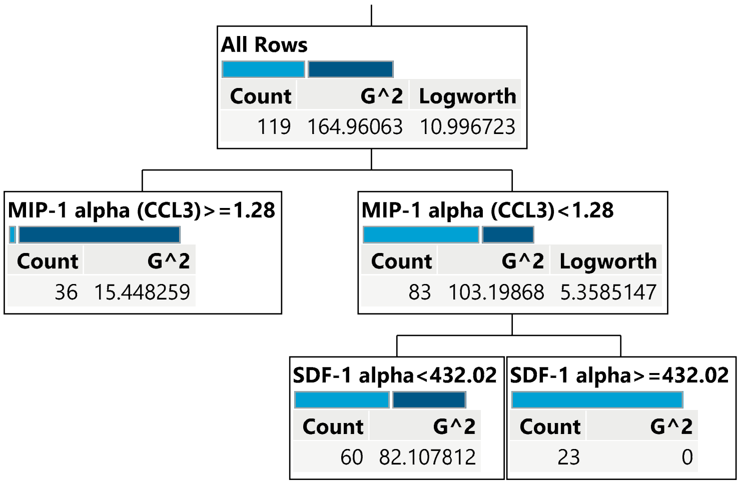

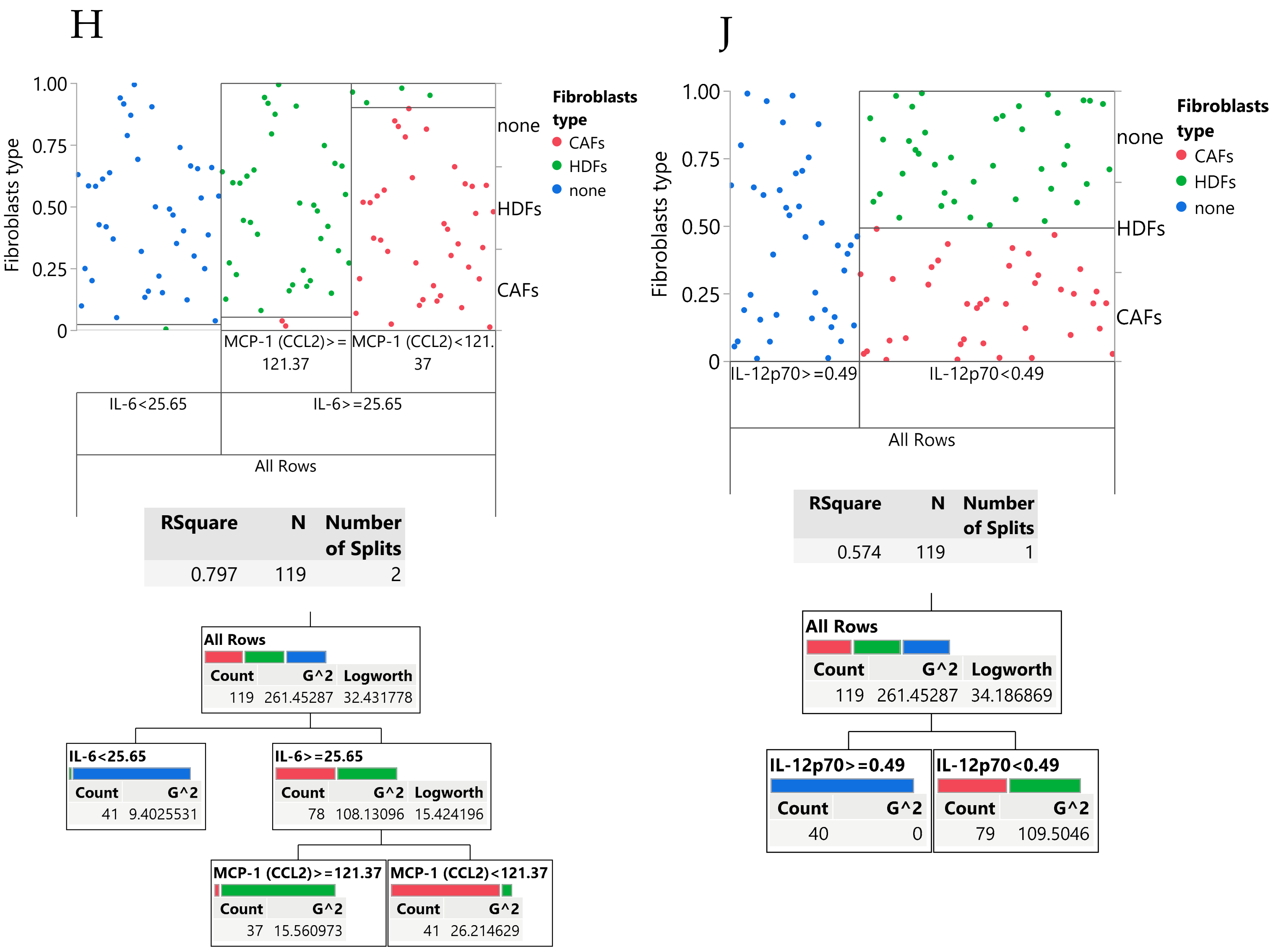


**Figure S4. The cytokine profile of the "high secretor" lines is positively influenced by the 3D culture setting and the presence of fibroblasts**. **A.** Wilcoxon Two-Sample Test analysis of Principal component 1 (PC1) and 2 (PC2) between the "high and low secretor" cell lines in 2D and 3D settings. **B.** Wilcoxon Two-Sample Test analysis of Principal component 1 (PC1) and 2 (PC2) between "high and low secretor"cell lines in 3D setting. **C.** Wilcoxon Two-Sample Test analysis of Principal component 1 (PC1) and 2 (PC2) between "high and low secretor" cell lines in 2D setting. **D.** Wilcoxon Two-Sample Test analysis of Principal component 1 (PC1) and 2 (PC2) between "high and low secretor" cell lines in coculture with CAFs. **E.** Wilcoxon Two-Sample Test analysis of Principal component 1 (PC1) and 2 (PC2) between "high and low secretor" cell lines in coculture with HDFs. **F.** Wilcoxon Two-Sample Test analysis of Principal component 1 (PC1) and 2 (PC2) between "high and low secretor" cell lines in monoculture. **G, H, J.** Partition plots and decision trees show the cytokines that explain at most the differences between the culture settings (G: 2D vs 3D; H, J: coculture with HDFs and CAFs vs monoculture).

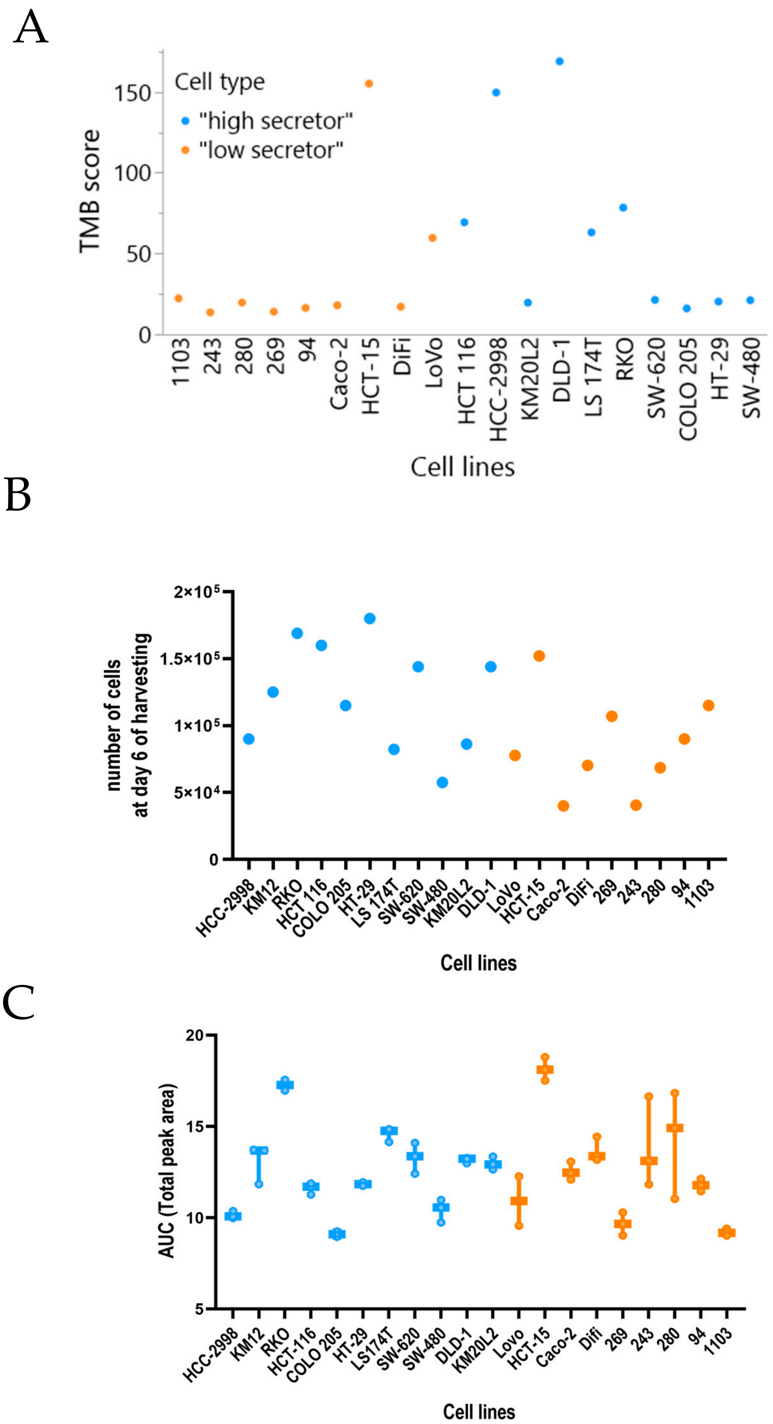


**Figure S5: TMB scores and proliferation rate of the CRC cell lines.** **A.** TMB scores of the CRC lines. **B.** Proliferation rate of the cancer cells in the 2D culture setting. The number of cells at day 6, i.e. day of harvesting of the supernatant for the cytokine analysis, are shown for the 20 CRC cell lines. **C.** Proliferation rate of the cancer cells in the 3D spheroids culture setting. AUC of the largest brightfield object areas (μm²) of the spheroids in monoculture for all 20 CRC cell lines are shown.

**Table S1:** Metadata about CRC cell lines: name, 2D and 3D assay doubling times, type of cell line based on cluster analysis, patient histology, gender, patient age at surgery, pathogenic status, site of origin.

| Cell lines | 2D doubling time [h] | 3D assay doubling time[h] | high/low secretor | Patient histology | Gender | Patient Age at Surgery | Pathogenic status | Site of origin |
|---|---|---|---|---|---|---|---|---|
| COLO 205 | 25 | 9 | "high secretor" | adeno carcinoma | male | 70 | Primary | Colon |
| DLD-1 | 20 | 13 | "high secretor" | adeno carcinoma | male | 20 | Primary | Colon |
| HCC-2998 | 32 | 10 | "high secretor" | adeno carcinoma | n.k. | n.k. | Primary | Colon |
| HCT 116 | 18 | 12 | "high secretor" | adeno carcinoma | male | n.k. | Primary | Colon |
| HT-29 | 16 | 12 | "high secretor" | adeno carcinoma | female | 44 | Primary | Colon |
| KM12 | 23 | 13 | "high secretor" | adeno carcinoma | n.k. | n.k. | Metastasis | Lymph Node |
| KM20L2 | 33 | 13 | "high secretor" | adeno carcinoma | n.k. | n.k. | Metastasis | Lung |
| LS 174T | 35 | 15 | "high secretor" | adeno carcinoma | female | 58 | Primary | Colon |
| RKO | 17 | 17 | "high secretor" | squamous carcinoma | n.k. | n.k. | Primary | Colon |
| SW-480 | 50 | 10 | "high secretor" | adeno carcinoma | male | 51 | Metastasis | Colon |
| SW-620 | 20 | 13 | "high secretor" | adeno carcinoma | male | 50 | Primary | Lymph Node |
| 94 | 32 | 12 | "low secretor" | adeno carcinoma | male | 70 | Primary | Colon |
| 243 | 71 | 14 | "low secretor" | adeno carcinoma | male | 45 | Metastasis | Liver |
| 269 | 27 | 10 | "low secretor" | adeno carcinoma | male | 56 | Primary | Colon |
| 280 | 42 | 14 | "low secretor" | adeno carcinoma | female | 55 | Metastasis | Skin |
| 1103 | 25 | 9 | "low secretor" | adeno carcinoma | male | 55 | Metastasis | Skin |
| Caco-2 | 72 | 13 | "low secretor" | adeno carcinoma | male | 72 | Primary | Colon |
| DiFi | 41 | 14 | "low secretor" | adeno carcinoma | female | 46 | Metastasis | Ascites |
| HCT-15 | 19 | 18 | "low secretor" | adeno carcinoma | male | n.k. | Primary | Colon |
| LoVo | 37 | 11 | "low secretor" | adeno carcinoma | male | 56 | Metastasis | metastatic nodule |
